# Local Well-Posedness to the Cauchy Problem for an Equation of the Nagumo Type

**DOI:** 10.1155/2022/5891265

**Published:** 2022-05-27

**Authors:** Vladimir Lizarazo, Richard De la cruz, Julio Lizarazo

**Affiliations:** ^1^School of Geological Engineering, Universidad Pedagógica y Tecnológica de Colombia, Calle 4 Sur 15-134, Sogamoso, Colombia; ^2^School of Mathematics and Statistics, Universidad Pedag´ogica y Tecnol´ogica de Colombia, Av. Central Del Norte 39-115, Tunja, Colombia

## Abstract

In this paper, we show the local well-posedness for the Cauchy problem for the equation of the Nagumo type in this equation (1) in the Sobolev spaces *H*^*s*^(*ℝ*). If *D* > 0, the local well-posedness is given for *s* > 1/2 and for *s* > 3/2 if *D*=0.

## 1. Introduction

In this paper, we show the local well-posedness for the following Cauchy problem:

(1) $$\begin{array}{c} \left\{\begin{array}{c} u_{t}=Du_{xx}-u\left(u-\alpha \right)\left(u-1\right)-\epsilon u^{n}u_{x},\;x\in \mathbb{R},t>0,\\ u\left(x,0\right)=\psi \left(x\right), \end{array}\right. \end{array}$$

where *D* > 0 is a constant diffusion coefficient, *α* ∈ (0, 1/2) and *ϵ* > 0 is a small positive quantity. In [1], the equation (1) was used to model chemotaxis (see equation (55) in [1]). Organisms which use chemotaxis to locate food sources include amoebae of the cellular slime mold *Dictyostelium discoideum*, and the motile bacterium *Escherichia coli* [1]. Therefore, *u*=*u*(*x*, *t*) models the population density, *n* is a positive integer, and *α* is a parameter which determines the minimal required density for a population to be able to survive (for normalized population density, i.e., such that *u*=1 is the maximum sustainable population). Balasuriya and Gottwald [1] studied the wave speed of travelling waves for the equation (1). Also, they have the numerical evidence for the wave speed of travelling waves for the equation (1). Other results related to the equation (1) can be found in [2].

When *ϵ*=0, the equation (1) is called a *Nagumo equation* or *bistable equation* [3–7] in which case the model describes an active pulse transmission line simulating a nerve axon.

Also, we can see the equation (1) as a generalized viscous Burgers equation with a source term. Dix [8] proved local well-posedness of the viscous Burgers equation with a source term using a contraction mapping argument. Moreover, for the classical Burgers equation (without viscosity) is well known that classical solutions cannot exits for all time, but weak global solutions can be established [9]. In addition, the uniqueness of the weak solution depends on some entropy condition. Observe that when *D*=0, the equation (1) is a generalized Burgers equation (without viscosity) and nonlinear source term. Therefore, from the mathematical viewpoint, the case *D*=0 is very interesting to study the existence and uniqueness of classical solution.

In this paper, we show the local well-posedness for the Cauchy problem to the equation of the Nagumo type (1) in the Sobolev spaces *H*^*s*^(*ℝ*) for *s* > 1/2 if *D* > 0, and for *s* > 3/2 if *D*=0. Our proof of local well-posedness is based on the results given in [10–12]. We use the Banach fixed point in a suitable complete space to guarantee the existence of local solutions to the problem (1) with *D* > 0. The Banach fixed point technique has been widely used to show existence and uniqueness of solutions to differential equations in Banach spaces (for instance, see [10–14] for more details). When *D*=0, we use the parabolic regularization method to show local well-posedness for the Cauchy problem (1) (e.g., [12,15]).

We will use the following notation: *ℝ* for the real numbers; *𝒮*(*ℝ*) for the Schwartz's space usual; $\hat{f}$ denotes the Fourier transform of *f*; the inverse Fourier transform will be denoted by ∨; by *H*^*s*^(*ℝ*), *s* ∈ *ℝ*, the set of all *f* ∈ *𝒮*′(*ℝ*) such that ${\left(1+\xi^{2}\right)}^{s/2}\hat{f}\in L^{2}\left(\mathbb{R}\right)$. *H*^*s*^(*ℝ*) is called the Sobolev space and it is a Hilbert space with respect to the inner product ${\left(f,g\right)}_{s}=\int_{\mathbb{R}}{\left(1+\xi^{2}\right)}^{s}\hat{f}\left(\xi \right)\bar{\hat{g}\left(\xi \right)}d\xi$; *C*(*I*; *X*) for the space of all continuous functions on an interval *I* into the Banach space *X*; if *I* is compact, *C*(*I*; *X*) is seen as a Banach space with the sup norm; *C*_*w*_(*I*; *X*) for the space of all weakly continuous functions on an interval *I* into Banach space *X*; *C*_*w*_^1^(*I*; *X*) for the space of all weakly differentiable functions on an interval *I* into Banach space *X*. We also denote by *V*(*t*)=*e*^*t*(*D∂*_*x*_^2^ − *α*Id)^, *t* ≥ 0, the semigroup in *H*^*s*^(*ℝ*) generated by the operator *tQ* where *Q*=(*D∂*_*x*_^2^ − *α*Id), i.e.,

(2) $$\begin{array}{c} V\left(t\right)f={\left(e^{-t\left(D\xi^{2}+\alpha \right)}\hat{f}\right)}^{\vee },\text{ for }f\in H^{s}\left(\mathbb{R}\right),t\geq 0, \end{array}$$

{*V*(*t*)}_*t*≥0_ is a *C*^0^-semigroup of contractions in *H*^*s*^(*ℝ*), *s* ∈ *ℝ*. Moreover, *u*(*x*, *t*)=*V*(*t*)*ψ*(*x*) is the unique solution to the linear problem associated with (1), i.e., *u*(*x*, *t*)=*V*(*t*)*ψ*(*x*) is the unique solution to the following problem.

(3) $$\begin{array}{c} \left\{\begin{array}{c} u_{t}=Du_{xx}-\alpha u,\;x\in \mathbb{R},t>0,\\ u\left(x,0\right)=\psi \left(x\right). \end{array}\right. \end{array}$$

**Proposition 1 .**

Let *ψ* ∈ *H*^*s*^(*ℝ*), *s* ∈ *ℝ*, *λ* ≥ 0, *D* > 0 and *t* > 0. Then, there exists a constant *C*_*λ*_, depending only on *λ*, such that

(4) $$\begin{array}{c} {\left\|V\left(t\right)\psi \right\|}_{s+\lambda }\leq C_{\lambda }{\left(1+{\left(\frac{\lambda }{2Dt}\right)}^{\lambda }\right)}^{1/2}{\left\|\psi \right\|}_{s}. \end{array}$$

In particular, *V*(*t*)*ψ* ∈ *𝒮*(*ℝ*) for all *t* > 0.

When there is no risk of confusion, we will use the notations *u*(*t*) for *u*(*x*, *t*), *ϕ* for *ϕ*(*x*), and *F*(*u*)=*ϵu*^*n*^*u*_*x*_+*u*^3^ − (*α*+1)*u*^2^.

## 2. Local Well-Posedness of the Problem (1) with *D* > 0

In this section, we use the Banach fixed point in a suitable complete metric space to show the existence of local solutions for integral equation (9) in Sobolev space *H*^*s*^(*ℝ*) for *s* > 1/2. In addition, the uniqueness of the solution and continuous dependence are established.

**Proposition 2 .**

Let *s* > 1/2 be fixed. Then, *F*(*u*) is a continuous map from *H*^*s*^(*ℝ*) into *H*^*s*−1^(*ℝ*) and satisfies the estimates as follows:

(5) $$\begin{array}{c} {\left\|F\left(u\right)-F\left(w\right)\right\|}_{s-1}\leq L_{s}\left({\left\|u\right\|}_{s},{\left\|w\right\|}_{s}\right){\left\|u-w\right\|}_{s}, \end{array}$$

for all *u*, *v* ∈ *H*^*s*^(*ℝ*), where *L*_*s*_(·, ·) is a continuous function, nondecreasing with respect to each of their arguments. In particular,

(6) $$\begin{array}{c} {\left\|F\left(u\right)\right\|}_{s-1}\leq L_{s}\left({\left\|u\right\|}_{s},0\right){\left\|u\right\|}_{s}. \end{array}$$

**Proof**

Observe that *F*(*u*)=(*ϵ*/*n*+1)(*u*^*n*+1^)_*x*_+*u*^3^ − (*α*+1)*u*^2^. Then, as *H*^*s*^(*ℝ*) is a Banach algebra for *s* > 1/2, we have the following:

(7) $$\begin{array}{c} {\left\|F\left(u\right)-F\left(w\right)\right\|}_{s-1}\leq \frac{\epsilon }{n+1}{\left\|\partial_{x}\left(u^{n+1}-w^{n+1}\right)\right\|}_{s-1}+{\left\|u^{3}-w^{3}\right\|}_{s-1}+\left(\alpha +1\right){\left\|u^{2}-w^{2}\right\|}_{s-1}\\ \leq \frac{\epsilon }{n+1}{\left\|u^{n+1}-w^{n+1}\right\|}_{s}+{\left\|u^{3}-w^{3}\right\|}_{s}+\left(\alpha +1\right){\left\|u^{2}-w^{2}\right\|}_{s}\\ =L_{s}\left({\left\|u\right\|}_{s},{\left\|w\right\|}_{s}\right){\left\|u-w\right\|}_{s}, \end{array}$$

where

(8) $$\begin{array}{c} L_{s}\left({\left\|u\right\|}_{s},{\left\|w\right\|}_{s}\right)=\frac{\epsilon }{n+1}\sum_{k=0}^{n}{\left\|u\right\|}_{s}^{n-k}{\left\|w\right\|}_{s}^{k}+\sum_{k=0}^{2}{\left\|u\right\|}_{s}^{n-k}{\left\|w\right\|}_{s}^{k}+\left(\alpha +1\right)\left({\left\|u\right\|}_{s}+{\left\|w\right\|}_{s}\right). \end{array}$$

The following result is to prove the existence of solutions. The proof is based in standard arguments [10,11]. We only present a sketch of proof.

**Proposition 3 .**

Let *D* > 0 be fixed, *s* > 1/2, *ψ* ∈ *H*^*s*^(*ℝ*), and *V*(*t*) is defined by (2). Then, there exists *T*=*T*(‖*ψ*‖_*s*_, *M*) > 0 and a unique function *u* ∈ *C*([0, *T*]; *H*^*s*^(*ℝ*)) satisfying the following integral equation:

(9) $$\begin{array}{c} u\left(\cdot ,t\right)=V\left(t\right)\psi \left(\cdot \right)-\int_{0}^{t}V\left(t-\tau \right)F\left(u\left(\cdot ,\tau \right)\right)d\tau . \end{array}$$

**Proof**

Let *M*, *T* > 0 be fixed, but arbitrary. Consider the following:

(10) $$\begin{array}{c} X\left(M,T,\psi \right)=\left\{u\in C\left(\left[0,T\right];H^{s}\left(\mathbb{R}\right)\right):\underset{\left[0,T\right]}{\sup}{\left\|u\left(t\right)-V\left(t\right)\psi \right\|}_{s}\leq M\right\}, \end{array}$$

which is a complete metric space with distance *d*(*u*, *v*)=sup_[0, *T*]_‖*u*(*t*) − *v*(*t*)‖_*s*_. Define on the space *𝒳*(*M*, *T*, *ψ*) the following map:

(11) $$\begin{array}{c} \left(Ag\right)\left(t\right)≔V\left(t\right)\psi -\int_{0}^{t}V\left(t-\tau \right)F\left(g\left(\tau \right)\right)d\tau . \end{array}$$

We have the following:

1. If *g* ∈ *𝒳*(*M*, *T*, *ψ*) then *𝒜g* ∈ *𝒳*(*M*, *T*, *ψ*)
2. We can choose $\tilde{T}>0$ sufficiently small such that $𝒜\left(𝒳\left(M,\tilde{T},\psi \right)\right)\subset 𝒳\left(M,\tilde{T},\psi \right)$
3. There exists $\bar{T}\in \left(0,\tilde{T}\right]$ such that *𝒜* is a contraction on $𝒳\left(M,\bar{T},\psi \right)$

So, *𝒜* has a unique fixed point *u* in $𝒳\left(M,\bar{T},\psi \right)$ which satisfies the integral equation (40) where $\bar{T}=T\left({\left\|\psi \right\|}_{s},M\right)>0$.

**Proposition 4 .**

The problem (1) is equivalent to the integral equation (40). More precisely, if *s* > 1/2 and *u* ∈ *C*([0, *T*]; *H*^*s*^(*ℝ*))∩*C*^1^((0, *T*]; *H*^*s*−2^(*ℝ*)) is a solution of (1), then *u* satisfies the integral equation (40). Conversely, if *s* > 1/2 and *u* ∈ *C*([0, *T*]; *H*^*s*^(*ℝ*)) is a solution of (40) then *u* ∈ *C*^1^([0, *T*]; *H*^*s*−2^(*ℝ*)) and satisfies (1).

**Proof**

Assume that *u* ∈ *C*([0, *T*]; *H*^*s*^(*ℝ*))∩*C*^1^((0, *T*]; *H*^*s*−2^(*ℝ*)) is a solution of (1). Then, (*d*/*dτ*)(*V*(*t* − *τ*)*u*(*τ*))=−*V*(*t* − *τ*)*F*(*u*(*τ*)), 0 < *τ* < *t*. So, *u* satisfies the integral equation (40). Conversely, assume that *u* ∈ *C*([0, *T*]; *H*^*s*^(*ℝ*)) is a solution of (40). For *t* > 0, let *η*(*t*) : −∫_0_^*t*^*V*(*t* − *τ*)*F*(*u*(*τ*))*dτ*. Then, for *h* > 0 arbitrary,

(12) $$\begin{array}{c} {\left\|\frac{\eta \left(t+h\right)-\eta \left(t\right)}{h}-Q\eta \left(t\right)+F\left(u\left(t\right)\right)\right\|}_{s-2}\leq \int_{0}^{t}{\left\|V\left(t-\tau \right)\left(\frac{V\left(h\right)-1}{h}-Q\right)F\left(u\left(\tau \right)\right)\right\|}_{s-2}d\tau \\ +\frac{1}{h}\int_{t}^{t+h}{\left\|V\left(t+h-\tau \right)F\left(u\left(\tau \right)\right)-F\left(u\left(t\right)\right)\right\|}_{s-2}d\tau . \end{array}$$

However,

(13) $$\begin{array}{c} {\left\|V\left(t-\tau \right)\left(\frac{V\left(h\right)-1}{h}-Q\right)F\left(u\left(\tau \right)\right)\right\|}_{s-2}\\ \leq C_{1}{\left(\frac{2D\left(t-\tau \right)+1}{2D\left(t-\tau \right)}\right)}^{1/2}{\left\|\left(\frac{V\left(h\right)-1}{h}-Q\right)F\left(u\left(\tau \right)\right)\right\|}_{s-2}\\ \leq C_{1}{\left(\frac{2D\left(t-\tau \right)+1}{2D\left(t-\tau \right)}\right)}^{1/2}K{\left\|F\left(u\left(\tau \right)\right)\right\|}_{s-2}\\ \leq C_{1}{\left(\frac{2D\left(t-\tau \right)+1}{2D\left(t-\tau \right)}\right)}^{1/2}K{\left\|F\left(u\left(\tau \right)\right)\right\|}_{s-1}\\ \leq C_{1}{\left(\frac{2D\left(t-\tau \right)+1}{2D\left(t-\tau \right)}\right)}^{1/2}KL_{s}\left(\underset{\tau \in \left[0,T\right]}{\sup}{\left\|u\left(\tau \right)\right\|}_{s},0\right)\underset{\tau \in \left[0,T\right]}{\sup}{\left\|u\left(\tau \right)\right\|}_{s}, \end{array}$$

and the right hand side of (57) is a integrable function of *τ* in [0, *t*]. Thus, using the dominated convergence theorem, we have as follows:

(14) $$\begin{array}{c} \lim_{h⟶0}+\int_{0}^{t}{\left\|V\left(t-\tau \right)\left(\frac{V\left(h\right)-1}{h}-Q\right)F\left(u\left(\tau \right)\right)\right\|}_{s-2}d\tau =0. \end{array}$$

Now, from the mean value theorem for integrals, there exists a value *c* on the interval (*t*, *t*+*h*) such that

(15) $$\begin{array}{c} \frac{1}{h}\int_{t}^{t+h}{\left\|V\left(t+h-\tau \right)F\left(u\left(\tau \right)\right)-F\left(u\left(t\right)\right)\right\|}_{s-2}d\tau ={\left\|V\left(t+h-c\right)F\left(u\left(c\right)\right)-F\left(u\left(t\right)\right)\right\|}_{s-2}\\ ={\left\|V\left(h-c^{*}\right)F\left(u\left(t+c^{*}\right)\right)-F\left(u\left(t\right)\right)\right\|}_{s-2}, \end{array}$$

and therefore, lim_*h*⟶0_+(1/*h*)∫_*t*_^*t*+*h*^‖*V*(*t*+*h* − *τ*)*F*(*u*(*τ*)) − *F*(*u*(*t*))‖_*s*−2_*dτ*=0.

After, *∂*_*t*_^+^*η*(*t*)=*Qη*(*t*) − *F*(*u*(*t*)) in *H*^*s*−2^(*ℝ*). where *∂*_*t*_^+^ is the right derivative. In similar way, we can conclude that the left derivative is *∂*_*t*_^−^*η*(*t*)=*Qη*(*t*) − *F*(*u*(*t*)) in *H*^*s*−2^(*ℝ*). So, *η* ∈ *C*^1^((0, *T*]; *H*^*s*−2^(*ℝ*)) and *∂*_*t*_*η*(*t*)=*Qη*(*t*) − *F*(*u*(*t*)). As *V*(*t*)*ψ*(*x*) is the solution of the linear problem (3), we conclude that *u*(*t*)=*V*(*t*)*ψ*+*η*(*t*) ∈ *C*^1^((0, *T*]; *H*^*s*−2^(*ℝ*)) and satisfies (1).

**Lemma 1 .**

Suppose *β* > 0, *γ* > 0, *β*+*γ* > 1, *a* ≥ 0, *b* ≥ 0, *u* is nonnegative and *t*^*γ*−1^*u*(*t*) is locally integrable on [0, *T*). If

(16) $$\begin{array}{c} u\left(t\right)\leq a+b\int_{0}^{t}{\left(t-s\right)}^{\beta -1}s^{\gamma -1}u\left(s\right)ds, \end{array}$$

i.e., in (0, *T*), then

(17) $$\begin{array}{c} u\left(t\right)\leq aE_{\beta ,\gamma }\left({\left(b\Gamma \left(\beta \right)\right)}^{1/\nu }t\right), \end{array}$$

where *ν*=*β*+*γ* − 1 > 0, *E*_*β*,*γ*_(*s*)=∑_*m*=0_^*∞*^*c*_*m*_*s*^*mν*^ with *c*_0_=1 and *c*_*m*+1_/*c*_*m*_=Γ(*mν*+*γ*)/Γ(*mν*+*γ*+*β*) for *m* ≥ 0.

The proof of this lemma is given in Lemma 7.1.2 in [16].

**Proposition 5 .**

Let *ψ*, *ϕ* ∈ *H*^*s*^(*ℝ*) and *u*, *v* ∈ *C*([0, *T*]; *H*^*s*^(*ℝ*)) be the corresponding solutions of equation (9). If *s* > 1/2, then

(18) $$\begin{array}{c} {\left\|u\left(t\right)-v\left(t\right)\right\|}_{s}\leq K{\left\|\psi -\phi \right\|}_{s}, \end{array}$$

where *K*=*E*_1/2,1_((*b*Γ(1/2))^2^*T*), $b=L_{s}\left(L,L\right)C_{1}\left(\sqrt{2DT}+1/\sqrt{2D}\right)$ and *L*=max{sup_[0, *T*]_‖*u*‖_*s*_, sup_[0, *T*]_‖*v*‖_*s*_} (here *E*_1/2,1_ is given by previous lemma).

**Proof**

Let *ψ*, *ϕ*, *u* and *v* as in the statement of the proposition. Let *s* > 1/2. From (9) we have as follows:

(19) $$\begin{array}{c} u\left(t\right)-v\left(t\right)=V\left(t\right)\left(\psi -\phi \right)-\int_{0}^{t}V\left(t-\tau \right)\left(F\left(u\left(\tau \right)\right)-F\left(v\left(\tau \right)\right)\right)d\tau . \end{array}$$

By Propositions 1 and 2, we obtain the following:

(20) $$\begin{array}{c} {\left\|u\left(t\right)-v\left(t\right)\right\|}_{s}\leq {\left\|\psi -\phi \right\|}_{s}+\int_{0}^{t}{\left\|V\left(t-\tau \right)\left(F\left(u\left(\tau \right)\right)-F\left(v\left(\tau \right)\right)\right)\right\|}_{s-1}d\tau \\ \leq {\left\|\psi -\phi \right\|}_{s}+C_{1}\int_{0}^{t}{\left(1+\frac{1}{2D\left(t-\tau \right)}\right)}^{1/2}{\left\|F\left(u\left(\tau \right)\right)-F\left(v\left(\tau \right)\right)\right\|}_{s-1}d\tau \\ \leq {\left\|\psi -\phi \right\|}_{s}+C_{1}\frac{\sqrt{2DT}+1}{\sqrt{2D}}\int_{0}^{t}{\left(t-\tau \right)}^{-1/2}{\left\|F\left(u\left(\tau \right)\right)-F\left(v\left(\tau \right)\right)\right\|}_{s-1}d\tau \\ \leq {\left\|\psi -\phi \right\|}_{s}+L_{s}\left(L,L\right)C_{1}\frac{\sqrt{2DT}+1}{\sqrt{2D}}\int_{0}^{t}{\left(t-\tau \right)}^{-1/2}{\left\|u\left(\tau \right)-v\left(\tau \right)\right\|}_{s}d\tau , \end{array}$$

where *L*=max{sup_[0, *T*]_‖*u*‖_*s*_, sup_[0, *T*]_‖*v*‖_*s*_}. Let $b=L_{s}\left(L,L\right)C_{1}\left(\sqrt{2DT}+1/\sqrt{2D}\right)$. Observe that *E*_1/2,1_((*b*Γ(1/2))^2^*T*) is finite. In fact, *E*_1/2,1_((*b*Γ(1/2))^2^*T*)=∑_*m*=0_^*∞*^*a*_*m*_ where *a*_*m*_=*c*_*m*_(*b*^2^*πT*)^*m*/2^. Therefore, (*a*_*m*+1_/*a*_*m*_)=(*c*_*m*+1_/*c*_*m*_)(*b*^2^*πT*)^1/2^ and from Lemma 1 we have that

(21) $$\begin{array}{c} \frac{c_{m+1}}{c_{m}}=\frac{\Gamma \left(\left(m/2\right)+1\right)}{\Gamma \left(\left(m+1/2\right)+1\right)}=\frac{m\Gamma \left(m/2\right)}{\left(m+1\right)\Gamma \left(m+1/2\right)}. \end{array}$$

As for all *x* > 0, $\Gamma \left(x\right)=\sqrt{2\pi }x^{x-\left(1/2\right)}e^{-x}e^{\theta \left(x\right)/12x}$ with 0 < *θ*(*x*) < 1, we have that 1 < *e*^*θ*(*x*)^ < *e* and *e*^(*m*/6)*θ*(*m*/2)−(6/*m*+1)*θ*(*m*+1/2)^ is bounded for *m* ≥ 1. From (21), we obtain as follows:

(22) $$\begin{array}{c} \frac{c_{m+1}}{c_{m}}=\frac{m}{m+1}\frac{{\left(m/2\right)}^{\left(m/2\right)-1}}{{\left(m+1/2\right)}^{\left(m+1/2\right)-1}}e^{-1/2}e^{\left(6/m\right)\theta \left(m/2\right)-\left(6/m+1\right)\theta \left(m+1/2\right)}⟶0,\text{as }m⟶\infty . \end{array}$$

So, lim_*m*⟶*∞*_|*a*_*m*+1_/*a*_*m*_|=0 and *E*_1/2,1_((*b*Γ(1/2))^2^*T*)=∑_*m*=0_^*∞*^*a*_*m*_, with *a*_*m*_=*c*_*m*_(*b*^2^*πT*)^*m*/2^, is convergent.

**Proposition 6 .**

Let *s* > 1/2. Then, the map *ψ* ↦ *u* is continuous in the following sense: if *ψ*_*n*_⟶*ψ* in *H*^*s*^(*ℝ*) and *u*_*n*_ ∈ *C*([0, *T*_*n*_]; *H*^*s*^(*ℝ*)), where *T*_*n*_=*T*(‖*ψ*_*n*_‖_*s*_, *M*) > 0, are the corresponding solutions (of the problem (1) with *u*_*n*_(0)=*ψ*_*n*_). Let *T* ∈ (0, *T*_*∞*_). Then, there exists a positive integer *N*=*N*(*D*, *ψ*, *T*) such that *T*_*n*_ ≥ *T* for all *n* ≥ *N* and

(23) $$\begin{array}{c} \lim_{n⟶\infty }\underset{\left[0,T\right]}{\sup}{\left\|u_{n}\left(t\right)-u\left(t\right)\right\|}_{s}=0. \end{array}$$

**Proof**

As *T*_*n*_=*T*(‖*ψ*_*n*_‖_*s*_, *M*) > 0 is a continuous function a ‖*ψ*_*n*_‖_*s*_, then there exists *N* ∈ *ℕ* such that *T*^*∗*^ ≤ *T*_*n*_ for all *n* ≥ *N*. Let *T*=min{*T*^*∗*^, *T*_1_, *T*_2_,…, *T*_*N*−1_}. Therefore, *u*_*n*_ is defined on [0, *T*] for all *n*. It follows that *u* ∈ *𝒳*(*M*, *T*, *ψ*_*n*_) for all *n* and satisfies ‖*u*_*n*_(*t*)‖_*s*_ ≤ ‖*ψ*_*n*_‖_*s*_+*M* ≤ *γ*+*M* where *γ*=sup_*n*_‖*ψ*_*n*_‖_*s*_. Therefore, sup_*t*∈[0, *T*]_‖*u*_*n*_(*t*)‖_*s*_ ≤ *γ*+*M* for all *n* and sup_*t*∈[0, *T*]_‖*u*(*t*)‖_*s*_ ≤ *γ*+*M*. Now, similar to the proof of the previous proposition, we have as follows:

(24) $$\begin{array}{c} {\left\|u_{n}\left(t\right)-u\left(t\right)\right\|}_{s}\leq {\left\|\psi_{n}-\psi \right\|}_{s}+L_{s}\left(\gamma +M,\gamma +M\right)C_{1}\\ \frac{\sqrt{2DT}+1}{\sqrt{2D}}\int_{0}^{t}{\left(t-\tau \right)}^{-1/2}{\left\|u_{n}\left(\tau \right)-u\left(\tau \right)\right\|}_{s}d\tau . \end{array}$$

Let $b=L_{s}\left(\gamma +M,\gamma +M\right)C_{1}\left(\sqrt{2DT}+1/\sqrt{2D}\right)$. Thus, *E*_1/2,1_((*b*Γ(1/2))^2^*T*) is finite (where *E*_1/2,1_ is given in Lemma 1) and we have as follows:

(25) $$\begin{array}{c} {\left\|u_{n}\left(t\right)-u\left(t\right)\right\|}_{s}\leq {\left\|\psi_{n}-\psi \right\|}_{s}E_{1/2,1}\left({\left(b\Gamma \left(\frac{1}{2}\right)\right)}^{2}T\right),\text{ for all }t\in \left[0,T\right]. \end{array}$$

This finishes the proof.

Finally, from Propositions 3, 5 and 6, we can summarize in the following theorem:

**Theorem 1 .**

Let *s* > 1/2. The problem (1) is locally well-posed in *H*^*s*^(*ℝ*).

## 3. Local Well-Posedness of the Problem (1) with *D* = 0

In this section, we show the local well-posedness of the problem (1) with *D*=0 using a priori estimate and the parabolic regularization method, the so-called vanishing viscosity method (for more details see [12]).

**Lemma 2 .**

Let *η*(*t*), *a*(*t*) and *b*(*t*) be real valued positive continuous functions defined on [0, *T*]⊆[0, +*∞*). Let *G*(*r*) and *H*(*r*) be positive continuous functions for *r* ≥ 0, with *G* strictly increasing and *H* nondecreasing. Define *A*(*t*)=sup_0≤*s*≤*t*_*a*(*s*) and *B*(*t*)=sup_0≤*s*≤*t*_*b*(*s*). Then, the inequality

(26) $$\begin{array}{c} G\left(\eta \left(t\right)\right)\leq a\left(t\right)+b\left(t\right)\int_{0}^{t}H\left(\eta \left(\tau \right)\right)d\tau ,\;0\leq t<T, \end{array}$$

implies the inequality

(27) $$\begin{array}{c} \eta \left(t\right)\leq G^{-1}\left(\Omega^{-1}\left(\Omega \left(A\left(t\right)\right)+tB\left(t\right)\right)\right),\;0\leq t\leq T_{*}\leq T, \end{array}$$

where Ω(*r*)=∫_*ϵ*_^*r*^(*dζ*/*H*(*G*^−1^(*ζ*))), *ϵ* > 0, *r* > 0, and *T*_*∗*_=sup{*τ* ∈ [0, *T*] : Ω(*A*(*τ*))+*τBτ*Ω(lim_*r*⟶*∞*_*G*(*r*))}.

**Proof**

This is a particular case of the theorem given in [17] [pp. 78].

**Proposition 7 .**

Let *s* > 3/2 be fixed. Then, *F*(*u*) satisfies the estimate

(28) $$\begin{array}{c} \left|{\left(u-w,F\left(u\right)-F\left(w\right)\right)}_{0}\right|\leq L_{0}\left({\left\|u\right\|}_{s},{\left\|w\right\|}_{s}\right){\left\|u-w\right\|}_{0}^{2}, \end{array}$$

for all *u*, *w* ∈ *H*^*s*^(*ℝ*), where *L*_0_(*x*, *y*)=*ϵx*∑_*k*=0_^*n*−1^*x*^*k*^*y*^*n*−1−*k*^+(*ϵn*/2)*y*^*n*^+*x*^2^+*xy*+*y*^2^+(*α*+1)(*x*+*y*).

**Proof**

We define *q*(*u*, *w*)=∑_*k*=0_^*n*−1^*u*^*k*^*w*^*n*−1−*k*^. As *s* > 3/2 thus *H*^*s*^(*ℝ*) and *H*^*s*−1^(*ℝ*) are Banach algebras. Moreover, we have that *H*^*s*^(*ℝ*)⟶*H*^*s*−1^(*ℝ*) and *H*^*s*^(*ℝ*)⟶*L*^*∞*^(*ℝ*). Thus, using the Cauchy–Schwartz inequality, we have as follows:

(29) $$\begin{array}{c} \left|{\left(u-w\left|F\left(u\right)-F\left(w\right)\right|\right)}_{0}\right|\leq \epsilon \left|{\left(u-w\left|\left(u^{n}-w^{n}\right)u_{x}\right|\right)}_{0}\right|+\epsilon \left|{\left(u-w\left|w^{n}{\left(u-w\right)}_{x}\right|\right)}_{0}\right|\\ +\left|{\left(u-w\left|\left(u^{3}-w^{3}\right)\right|\right)}_{0}\right|+\left(\alpha +1\right)\left|{\left(u-w\left|u^{2}-w^{2}\right|\right)}_{0}\right|\\ \leq \epsilon {\left\|u-w\right\|}_{0}{\left\|\left(u^{n}-w^{n}\right)u_{x}\right\|}_{0}+\frac{\epsilon }{2}\left|{\left(\left|{\left({\left(u-w\right)}^{2}\right)}_{x}\right|w^{n}\right)}_{0}\right|\\ +{\left\|u-w\right\|}_{0}{\left\|u^{3}-w^{3}\right\|}_{0}+\left(\alpha +1\right){\left\|u-w\right\|}_{0}{\left\|u^{2}-w^{2}\right\|}_{0}\\ \leq \left(\epsilon {\left\|u_{x}\right\|}_{L^{\infty }}{\left\|q\left(u,w\right)\right\|}_{L^{\infty }}+\frac{\epsilon }{2}\left|{\left(\left|{\left(u-w\right)}^{2}\right|{\left(w^{n}\right)}_{x}\right)}_{0}\right|+{\left\|u^{2}+uw+w^{2}\right\|}_{L^{\infty }}+\left(\alpha +1\right){\left\|u+w\right\|}_{L^{\infty }}\right)\\ {\left\|u-w\right\|}_{0}^{2}\\ \leq \left(\epsilon {\left\|u_{x}\right\|}_{s-1}{\left\|q\left(u,w\right)\right\|}_{s-1}+\frac{\epsilon }{2}{\left\|{\left(w^{n}\right)}_{x}\right\|}_{L^{\infty }}+{\left\|u^{2}+uw+w^{2}\right\|}_{s-1}+\left(\alpha +1\right){\left\|u+w\right\|}_{s-1}\right){\left\|u-w\right\|}_{0}^{2}\\ \leq \left(\epsilon {\left\|u\right\|}_{s}{\left\|q\left(u,w\right)\right\|}_{s}+\frac{\epsilon n}{2}{\left\|w^{n-1}w_{x}\right\|}_{L^{\infty }}+{\left\|u^{2}+uw+w^{2}\right\|}_{s-1}+\left(\alpha +1\right){\left\|u+w\right\|}_{s-1}\right)\\ {\left\|u-w\right\|}_{0}^{2}\\ \leq \left(\epsilon {\left\|u\right\|}_{s}{\left\|q\left(u,w\right)\right\|}_{s}+\frac{\epsilon n}{2}{\left\|w^{n-1}\right\|}_{s-1}{\left\|w_{x}\right\|}_{s-1}+{\left\|u^{2}+uw+w^{2}\right\|}_{s}+\left(\alpha +1\right){\left\|u+w\right\|}_{s}\right)\\ {\left\|u-w\right\|}_{0}^{2}\leq L_{0}\left({\left\|u\right\|}_{s},{\left\|v\right\|}_{s}\right){\left\|u-w\right\|}_{0}^{2}. \end{array}$$

**Lemma 3 .**

(T. Kato). Let *r* ≥ 1 and *s* > 3/2 be fixed and *h*, *v* are real valued functions. Then, there exists a constant *C*=*C*(*r*, *s*) such that

(30) $$\begin{array}{c} \left|{\left(v,h\partial_{x}v\right)}_{r}\right|\leq C\left({\left\|\partial_{x}h\right\|}_{s-1}{\left\|v\right\|}_{r}^{2}+{\left\|\partial_{x}h\right\|}_{r-1}{\left\|v\right\|}_{r}{\left\|v\right\|}_{s}\right). \end{array}$$

In particular, |(*v*, *h∂*_*x*_*v*)_*s*_| ≤ *C*‖*∂*_*x*_*h*‖_*s*−1_‖*v*‖_*s*_^2^.

**Proof**

See Lemma A.5. in [13].

**Theorem 2 .**

Let *s* > 3/2 be fixed. For *D* > 0, consider the initial value problem (1) with initial data + and let *u*_*D*_ ∈ *C*([0, *T*_*s*_^*∗*^]; *H*^*s*^(*ℝ*)) be the corresponding solution of (1) for some *T*_*s*_^*∗*^ > 0. Then, there exists a *T*_*s*_=*T*_*s*_(*ψ*) > 0, depending on ‖*ψ*‖_*s*_, such that *u*_*D*_ can be extended to the interval [0, *T*_*s*_(*ψ*)], and there is a function *ρ* ∈ *C*([0, *T*_*s*_(*ψ*)]; [0, +*∞*)) such that *ρ*(0)=‖*ψ*‖_*s*_^2^ and ‖*u*_*D*_(*t*)‖_*s*_^2^ ≤ *ρ*(*t*),  for all *t* ∈ [0, *T*_*s*_(*ψ*)].

**Proof**

Using the inner product in *H*^*s*^(*ℝ*) and Lemma 3 we have that

(31) $$\begin{array}{c} \partial_{t}{\left\|u_{D}\right\|}_{s}^{2}=2{\left(u_{D}\left|\partial_{t}u_{D}\right|\right)}_{s}=2{\left(u_{D}\left|D\partial_{x}^{2}u_{D}-u_{D}\left(u_{D}-\alpha \right)\left(u_{D}-1\right)-\epsilon u_{D}^{n}\partial_{x}u_{D}\right|\right)}_{s}\\ =2\left[{\left(u_{D}\left|D\partial_{x}^{2}u_{D}\right|\right)}_{s}+{\left(u_{D}\left|-u_{D}\left(u_{D}-\alpha \right)\left(u_{D}-1\right)\right|\right)}_{s}+{\left(u_{D}\left|-\epsilon u_{D}^{n}\partial_{x}u_{D}\right|\right)}_{s}\right]\\ =2\left[-D{\left\|\partial_{x}u_{D}\right\|}_{s}^{2}-{\left(u_{D}\left|u_{D}\left(u_{D}-\alpha \right)\left(u_{D}-1\right)\right|\right)}_{s}-\epsilon {\left(u_{D}\left|u_{D}^{n}\partial_{x}u_{D}\right|\right)}_{s}\right]\\ \leq 2C_{s}\left({\left\|u_{D}\right\|}_{s}^{4}+\left(\alpha +1\right){\left\|u_{D}\right\|}_{s}^{3}+\epsilon {\left\|\partial_{x}u_{D}^{n}\right\|}_{s-1}{\left\|u_{D}\right\|}_{s}^{2}\right)\\ \leq 2C_{s}\left({\left\|u_{D}\right\|}_{s}^{4}+\left(\alpha +1\right){\left\|u_{D}\right\|}_{s}^{3}+\epsilon {\left\|u_{D}\right\|}_{s}^{n+2}\right),\text{ for all }t\in \left(0,T_{s}^{*}\left(D,\psi \right)\right). \end{array}$$

Then, ‖*u*_*D*_(*t*)‖_*s*_^2^ ≤ *ρ*(*t*) for all *t* ∈ [0, *T*^*∗*^), where *ρ* ∈ *C*([0, *T*^*∗*^); [0, +*∞*)) is the maximally extended solution of the following problem.

(32) $$\begin{array}{c} \left\{\begin{array}{c} \frac{d\rho \left(t\right)}{dt}=2C_{s}\left(\rho^{2}+\left(\alpha +1\right)\rho^{3/2}+\epsilon \rho^{\left(n+2\right)/2}\right),\\ \rho \left(0\right)={\left\|\psi \right\|}_{s}^{2}. \end{array}\right. \end{array}$$

For *n* ≥ 2, from the problem (32) we obtain as follows:

(33) $$\begin{array}{c} \frac{d\rho \left(t\right)}{dt}=2C_{s}\left(\rho^{2}dt+\left(\alpha +1\right)\rho^{3/2}\left(t\right)+\epsilon \rho^{\left(n+2\right)/2}\left(t\right)\right)\leq 2C_{s}\left(\alpha +\epsilon +2+4\rho^{\left(n+2\right)/2}\left(t\right)\right), \end{array}$$

and integrating from 0 to *t* we have as follows:

(34) $$\begin{array}{c} \rho \left(t\right)\leq {\left\|\psi \right\|}_{s}^{2}+2C_{s}\left(\alpha +\epsilon +2\right)t+8C_{s}\int_{0}^{t}\rho^{\left(n+2\right)/2}\left(\tau \right)d\tau ,\;0\leq t\leq T<T^{*}. \end{array}$$

From Lemma 2 with *a*(*t*)=‖*ψ*‖_*s*_^2^+2*C*_*s*_(*α*+*ϵ*+2)*t*, *b*(*t*)=8*C*_*s*_, *G*(*r*)=*r* and *H*(*r*)=*r*^(*n*+2)/2^ we have the following bound:

(35) $$\begin{array}{c} \rho \left(t\right)\leq {\left({\left({\left\|\psi \right\|}_{s}^{2}+2C_{s}\left(\alpha +\epsilon +2\right)t\right)}^{-n/2}-4nC_{s}t\right)}^{-\left(2/n\right)}, \end{array}$$

for *t* ≤ *T*_*∗*_, where *T*_*∗*_=sup{*t* ∈ [0, +*∞*) : (‖*ψ*‖_*s*_^2^+2*C*_*s*_(*α*+*ϵ*+2)*t*)^−*n*/2^ ≥ 4*nC*_*s*_*t*}. Observe that *T*_*∗*_ > 0, since the function Φ(*t*)=(‖*ψ*‖_*s*_^2^+2*C*_*s*_(*α*+*ϵ*+2)*t*)^−*n*/2^ − 4*nC*_*s*_*t* is strictly decreasing for *t* ≥ 0, Φ(0)=(1/‖*ψ*‖_*s*_^*n*^) and there exists an unique *t*_*r*_ ∈ (0, +*∞*) such that Φ(*t*_*r*_)=0. Therefore, we can choose *T*_*s*_(*ψ*) such that 0 < *T*_*s*_(*ψ*) < *T*_*∗*_. Moreover, the function *W*(*t*)=(Φ(*t*))^−2/*n*^ is increasing on [0, *T*_*s*_(*ψ*)], and therefore we have that

(36) $$\begin{array}{c} \rho \left(t\right)\leq {\left({\left({\left\|\psi \right\|}_{s}^{2}+2C_{s}\left(\alpha +\epsilon +2\right)T_{s}\left(\psi \right)\right)}^{-n/2}-4nC_{s}T_{s}\left(\psi \right)\right)}^{-\left(2/n\right)}, \end{array}$$

for all *t* ∈ [0, *T*_*s*_(*ψ*)].

For the case *n*=1, from (32) we have that

(37) $$\begin{array}{c} \rho \left(t\right)\leq {\left\|\psi \right\|}_{s}^{2}+2C_{s}\left(\alpha +\epsilon +2\right)t+8C_{s}\int_{0}^{t}\rho^{2}\left(\tau \right)d\tau ,\;0\leq t\leq T<T^{*}, \end{array}$$

and from Lemma 2 we have *ρ*(*t*) ≤ (1/(‖*ψ*‖_*s*_^2^+2*C*_*s*_(*α*+*ϵ*+2)*t*)^−1^ − 8*C*_*s*_*t*), for 0 ≤ *t* ≤ *T*_*∗*_ where $T_{*}=\left(-{\left\|\psi \right\|}_{s}^{2}+\sqrt{{\left\|\psi \right\|}_{s}^{4}+4\left(\alpha +\epsilon +2\right)}/16C_{s}\left(\alpha +\epsilon +2\right)\right)$. So, we can choose *T*_*s*_(*ψ*) such that 0 < *T*_*s*_(*ψ*) < *T*_*∗*_, and therefore, we conclude that

(38) $$\begin{array}{c} \rho \left(t\right)\leq \frac{1}{{\left({\left\|\psi \right\|}_{s}^{2}+2C_{s}\left(\alpha +\epsilon +2\right)T_{s}\left(\psi \right)\right)}^{-1}-8C_{s}T_{s}\left(\psi \right)}, \end{array}$$

for all *t* ∈ [0, *T*_*s*_(*ψ*)].

As, ‖*u*_*D*_(*t*)‖_*s*_^2^ ≤ *ρ*(*t*) for all *t* ∈ [0, *T*^*∗*^), and since *ρ*(*t*) and *T*^*∗*^ do not depend on *D*, the usual extension method shows that we must have *T*_*s*_^*∗*^(*D*, *ψ*) ≥ *T*_*s*_(*ψ*) for all *D* > 0, where *T*_*s*_(*ψ*) is any positive number satisfying 0 < *T*_*s*_(*ψ*) < *T*^*∗*^.

**Theorem 3 .**

Let *s* > 3/2 be fixed. If *ψ* ∈ *H*^*s*^(*ℝ*), then there exists a *T*_*s*_=*T*_*s*_(*ψ*) and a function *u*_0_ ∈ *C*_*w*_([0, *T*_*s*_]; *H*^*s*^(*ℝ*))∩*C*_*w*_^1^([0, *T*_*s*_]; *H*^*s*−2^(*ℝ*)) such that *u*_0_(0)=*ψ*, and *u*_0_ satisfies (1) with *D*=0, in the weak sense, i.e.,

(39) $$\begin{array}{c} \frac{d}{dt}{\left(u_{0}\left(t\right)\left|\varphi \right|\right)}_{s-2}={\left(-\alpha u_{0}-\epsilon u_{0}^{n}\partial_{x}u_{0}-u_{0}^{3}+\left(\alpha +1\right)u_{0}^{2}\left|\varphi \right|\right)}_{s-2}, \end{array}$$

for all *φ* ∈ *H*^*s*−2^(*ℝ*) and *t* ∈ [0, *T*_*s*_].

Moreover, ‖*u*_0_‖_*s*_^2^ ≤ *ρ*(*t*) for all *t* ∈ [0, *T*_*s*_], where *ρ*(*t*) is as in Theorem 2.

**Proof**

Let *T*_*s*_=*T*_*s*_(*ψ*) be as in Theorem 2. Now, we will split the proof into four steps:

**Step 1 .**

First we will show that (*u*_*D*_(*t*))_*D*>0_ is a net which converges to a function *u*_0_ ∈ *C*([0, *T*_*s*_]; *L*^2^(*ℝ*)) in the *L*^2^− norm, uniformly over [0, *T*_*s*_].

Let *D*_1_, *D*_2_ ∈ (0, +*∞*). Then,

(40) $$\begin{array}{c} \frac{1}{2}\frac{d}{dt}{\left\|u_{D_{1}}-u_{D_{2}}\right\|}_{0}^{2}={\left(u_{D_{1}}-u_{D_{2}}\left|\frac{d}{dt}du_{D_{1}}-u_{D_{2}}\right|\right)}_{0}\\ ={\left(u_{D_{1}}-u_{D_{2}}\left|D_{1}\partial_{x}^{2}u_{D_{1}}-D_{2}\partial_{x}^{2}u_{D_{2}}\right|\right)}_{0}-{\left(u_{D_{1}}-u_{D_{2}}\left|u_{D_{1}}^{3}-u_{D_{2}}^{3}\right|\right)}_{0}\\ +\left(\alpha +1\right){\left(u_{D_{1}}-u_{D_{2}}\left|u_{D_{1}}^{2}-u_{D_{2}}^{2}\right|\right)}_{0}-\alpha {\left(u_{D_{1}}-u_{D_{2}}\left|u_{D_{1}}-u_{D_{2}}\right|\right)}_{0}-\epsilon {\left(u_{D_{1}}-u_{D_{2}}\left|u_{D_{1}}^{n}\partial_{x}u_{D_{1}}-u_{D_{2}}^{n}\partial_{x}u_{D_{2}}\right|\right)}_{0}\\ =-D_{1}{\left\|\partial_{x}\left(u_{D_{1}}-u_{D_{2}}\right)\right\|}_{0}^{2}-\left(D_{1}-D_{2}\right){\left(\partial_{x}\left(u_{D_{1}}-u_{D_{2}}\right)\left|\partial_{x}u_{D_{2}}\right|\right)}_{0}\\ -{\left(u_{D_{1}}-u_{D_{2}}\left|u_{D_{1}}^{3}-u_{D_{2}}^{3}\right|\right)}_{0}+\left(\alpha +1\right){\left(u_{D_{1}}-u_{D_{2}}\left|u_{D_{1}}^{2}-u_{D_{2}}^{2}\right|\right)}_{0}\\ -\alpha {\left\|u_{D_{1}}-u_{D_{2}}\right\|}_{0}^{2}-{\left(\epsilon \left(u_{D_{1}}-u_{D_{2}}\left|u_{D_{1}}^{n}\partial_{x}u_{D_{1}}-u_{D_{2}}^{n}\partial_{x}u_{D_{2}}\right|\right)\right)}_{0}\\ \leq \left|D_{1}-D_{2}\right|\left|{\left(\partial_{x}\left(u_{D_{1}}-u_{D_{2}}\right)\left|\partial_{x}u_{D_{2}}\right|\right)}_{0}\right|+\left|{\left(u_{D_{1}}-u_{D_{2}}\left|u_{D_{1}}^{3}-u_{D_{2}}^{3}\right|\right)}_{0}\right|\\ +\left(\alpha +1\right)\left|{\left(u_{D_{1}}-u_{D_{2}}\left|u_{D_{1}}^{2}-u_{D_{2}}^{2}\right|\right)}_{0}\right|\\ +\epsilon \left|{\left(\left(u_{D_{1}}-u_{D_{2}}\left|u_{D_{1}}^{n}\partial_{x}u_{D_{1}}-u_{D_{2}}^{n}\partial_{x}u_{D_{2}}\right|\right)\right)}_{0}\right|,\text{ for all }t\in \left[0,T_{s}\right]. \end{array}$$

Let $M=\sup_{t\in \left[0,T_{s}\right]}\sqrt{\rho \left(t\right)}$, where *ρ* is the function defined in the proof of Theorem 2. We bound separately each term on the right-hand side of (40) as follows:

In order to bound the first term, we have as follows:

(41) $$\begin{array}{c} \left|D_{1}-D_{2}{\left\|\left(\partial_{x}\left(u_{D_{1}}-u_{D_{2}}\right)\left|\partial_{x}\left(u_{D_{2}}\right)\right|\right)\right\|}_{0}\right|\leq \left|D_{1}-D_{2}\right|{\left\|\partial_{x}\left(u_{D_{1}}-u_{D_{2}}\right)\right\|}_{0}{\left\|\partial_{x}\left(u_{D_{2}}\right)\right\|}_{0}\\ \leq \left|D_{1}-D_{2}\right|\left({\left\|\partial_{x}u_{D_{1}}\right\|}_{0}+{\left\|\partial_{x}u_{D_{2}}\right\|}_{0}\right){\left\|\partial_{x}u_{D_{2}}\right\|}_{0}\\ \leq 2M^{2}\left|D_{1}-D_{2}\right|. \end{array}$$

We can bound the second term by the following:

(42) $$\begin{array}{c} \left|{\left(u_{D_{1}}-u_{D_{2}}\left|u_{D_{1}}^{3}-u_{D_{2}}^{3}\right|\right)}_{0}\right|\leq {\left\|u_{D_{1}}-u_{D_{2}}\right\|}_{0}^{2}\left({\left\|u_{D_{1}}\right\|}_{0}^{2}+{\left\|u_{D_{1}}\right\|}_{0}{\left\|u_{D_{2}}\right\|}_{0}+{\left\|u_{D_{2}}\right\|}_{0}^{2}\right)\\ \leq {\left\|u_{D_{1}}-u_{D_{2}}\right\|}_{0}^{2}\left({\left\|u_{D_{1}}\right\|}_{s}^{2}+{\left\|u_{D_{1}}\right\|}_{s}{\left\|u_{D_{2}}\right\|}_{s}+{\left\|u_{D_{2}}\right\|}_{s}^{2}\right)\\ \leq 3M^{2}{\left\|u_{D_{1}}-u_{D_{2}}\right\|}_{0}^{2}. \end{array}$$

The third term is bounded by |(*u*_*D*_1__ − *u*_*D*_2__|*u*_*D*_1__^2^ − *u*_*D*_2__^2^|)_0_| ≤ 2*M*‖*u*_*D*_1__ − *u*_*D*_2__‖_0_^2^.

Finally, we have

(43) $$\begin{array}{c} \left|{\left(u_{D_{1}}-u_{D_{2}}\left|u_{D_{1}}^{n}\partial_{x}u_{D_{1}}-u_{D_{2}}^{n}\partial_{x}u_{D_{2}}\right|\right)}_{0}\right|\\ =\frac{1}{n+1}\left|{\left(u_{D_{1}}-u_{D_{2}}\left|\partial_{x}\left(u_{D_{1}}^{n+1}-u_{D_{2}}^{n+1}\right)\right|\right)}_{0}\right|\\ =\frac{1}{n+1}\left|{\left(\partial_{x}\left(u_{D_{1}}-u_{D_{2}}\right)\left|u_{D_{1}}^{n+1}-u_{D_{2}}^{n+1}\right|\right)}_{0}\right|\\ =\frac{1}{n+1}\left|{\left(\partial_{x}\left(u_{D_{1}}-u_{D_{2}}\right)\left|\left(u_{D_{1}}-u_{D_{2}}\right)q\left(u_{D_{1}},u_{D_{2}}\right)\right|\right)}_{0}\right|\\ =\frac{1}{2\left(n+1\right)}\left|{\left({\left(u_{D_{1}}-u_{D_{2}}\right)}^{2}\left|\partial_{x}q\left(u_{D_{1}},u_{D_{2}}\right)\right|\right)}_{0}\right|\\ \leq \frac{1}{2\left(n+1\right)}{\left\|u_{D_{1}}-u_{D_{2}}\right\|}_{0}^{2}{\left\|\partial_{x}q\left(u_{D_{1}},u_{D_{2}}\right)\right\|}_{L^{\infty }}, \end{array}$$

(where *q* is defined in the proof of Proposition 7). From Theorem 2, we obtain as follows:

(44) $$\begin{array}{c} {\left\|\partial_{x}q\left(u_{D_{1}},u_{D_{2}}\right)\right\|}_{L^{\infty }}\leq {\left\|\partial_{x}q\left(u_{D_{1}},u_{D_{2}}\right)\right\|}_{s}\leq \rho {\left(t\right)}^{n/2}\leq M^{n}, \end{array}$$

for all *t* ∈ [0, *T*_*s*_].

Therefore, from the above bounds, we have as follows:

(45) $$\begin{array}{c} \frac{1}{2}\frac{d}{dt}{\left\|u_{D_{1}}-u_{D_{2}}\right\|}_{0}^{2}\leq 2M^{2}\left|D_{1}-D_{2}\right|+\left(3M^{2}+2\left(\alpha +1\right)M+\frac{M^{n}}{2\left(n+1\right)}\right){\left\|u_{D_{1}}-u_{D_{2}}\right\|}_{0}^{2}. \end{array}$$

Applying Gronwall's inequality to the last relation, we show that there is a constant *C* > 0 satisfying ‖*u*_*D*_1__(*t*) − *u*_*D*_2__(*t*)‖_0_^2^ ≤ *C*|*D*_1_ − *D*_2_| for all *t* ∈ [0, *T*_*s*_], and since *L*^2^(*ℝ*) is complete there exists the limit *u*_0_(*t*)=lim_*D*⟶0_*u*_*D*_(*t*) in *L*^2^(*ℝ*) uniformly with respect to *t* ∈ [0, *T*_*s*_], i.e.

(46) $$\begin{array}{c} \underset{D⟶0}{\lim\;}+\underset{t\in \left[0,T_{s}\right]}{\sup}{\left\|u_{D}\left(t\right)-u_{0}\left(t\right)\right\|}_{0}=0, \end{array}$$

and so *u*_0_ ∈ *C*([0, *T*_*s*_]; *L*^2^(*ℝ*)).

**Step 2 .**

Now we show that *u*_0_ ∈ *H*^*s*^(*ℝ*). Let *t* ∈ [0, *T*_*s*_]. Since *u*_*D*_⟶*u*_0_ in *L*^2^(*ℝ*), as *D*⟶0+, then there exists a subsequence {*D*_*n*_^(*j*)^} such that

(47) $$\begin{array}{c} \underset{j⟶\infty }{\lim\;}\hat{u_{D_{n}^{\left(j\right)}}}\left(t,\xi \right)=\hat{u_{0}}\left(t,\xi \right),\xi -\text{a.e.}. \end{array}$$

We obtain by Fatou's Lemma as follows:

(48) $$\begin{array}{c} {\left\|u_{0}\right\|}_{s}^{2}=\int_{\mathbb{R}}{\left(1+\xi^{2}\right)}^{s}{\left|\hat{u_{0}}\right|}^{2}d\xi \leq \underset{j⟶\infty }{\mathrm{liminf}}\int_{\mathbb{R}}{\left(1+\xi^{2}\right)}^{s}{\left|\hat{u_{D_{n}^{\left(j\right)}}}\right|}^{2}d\xi \leq \rho \left(t\right). \end{array}$$

**Step 3 .**

We must show that *u*_*D*_⇀*u*_0_ in *H*^*s*^(*ℝ*) for all *t* ∈ [0, *T*_*s*_] as *D*⟶0+.

First of all, we will show that (*u*_*D*_(*t*))_*D*>0_ is a weak Cauchy net in *H*^*s*^(*ℝ*), uniformly with respect to *t* ∈ [0, *T*_*s*_]. In fact, given *φ* ∈ *H*^*s*^(*ℝ*) and *ϵ* > 0, choosing *φ*_*ϵ*_ ∈ *H*^*s*^(*ℝ*) such that ‖*φ* − *φ*_*ϵ*_‖_*s*_ ≤ *ϵ*, then

(49) $$\begin{array}{c} \left|{\left(u_{D_{1}}\left(t\right)-u_{D_{2}}\left(t\right)\left|\varphi \right|\right)}_{s}\right|=\left|{\left(u_{D_{1}}\left(t\right)-u_{D_{2}}\left(t\right)\left|\varphi -\varphi_{\epsilon }\right|\right)}_{s}+{\left(u_{D_{1}}\left(t\right)-u_{D_{2}}\left(t\right)\left|\varphi_{\epsilon }\right|\right)}_{s}\right|\\ \leq {\left\|u_{D_{1}}\left(t\right)-u_{D_{2}}\left(t\right)\right\|}_{s}{\left\|\varphi -\varphi_{\epsilon }\right\|}_{s}+\left|{\left(u_{D_{1}}\left(t\right)-u_{D_{2}}\left(t\right)\left|{\left(1-\partial_{x}^{2}\right)}^{s}\varphi_{\epsilon }\right|\right)}_{0}\right|\\ \leq 2M\epsilon +{\left\|u_{D_{1}}\left(t\right)-u_{D_{2}}\left(t\right)\right\|}_{0}{\left\|{\left(1-\partial_{x}^{2}\right)}^{s}\varphi_{\epsilon }\right\|}_{0}\\ \leq 2M\epsilon +C\left|D_{1}-D_{2}\right|,\text{ for all }t\in \left[0,T_{s}\left(\psi \right)\right], \end{array}$$

and therefore, we have lim_*D*_1_,*D*_2_⟶0_+sup_*t*∈[0, *T*_*s*_]_(*u*_*D*_1__(*t*) − *u*_*D*_2__(*t*)|*φ*|)_*s*_=0.

Thus, we have that *u*_*D*_⇀*u*_0_ for all *t* ∈ [0, *T*], i.e.,

(50) $$\begin{array}{c} \lim_{D⟶0}\;+\underset{t\in \left[0,T_{s}\right]}{\sup}{\left(u_{D}\left(t\right)-u_{0}\left(t\right)\left|\varphi \right|\right)}_{s}=0, \end{array}$$

for all *φ* ∈ *H*^*s*^(*ℝ*). Moreover, since the convergence is uniform for all *φ* ∈ *H*^*s*^(*ℝ*), we can conclude that *u*_0_ ∈ *C*_*w*_([0, *T*_*s*_]; *H*^*s*^(*ℝ*)).

**Step 4 .**

Finally, we show that *u*_0_ ∈ *C*_*w*_^1^([0, *T*_*s*_]; *H*^*s*−2^(*ℝ*)).

Let *φ* ∈ *H*^*s*−2^(*ℝ*). Then,

(51) $$\begin{array}{c} {\left(u_{D}\left(t\right)\left|\varphi \right|\right)}_{s-2}={\left(\psi \left|\varphi \right|\right)}_{s-2}\\ +\int_{0}^{t}{\left(D\partial_{x}^{2}u_{D}\left(\tau \right)-u_{D}\left(\tau \right)\left(u_{D}\left(\tau \right)-\alpha \right)\left(u_{D}\left(\tau \right)-1\right)-\epsilon u_{D}{\left(\tau \right)}^{n}\partial_{x}\left(u_{D}\left(\tau \right)\right)\left|\varphi \right|\right)}_{s-2}d\tau , \end{array}$$

for all *t* ∈ [0, *T*_*s*_]. Since *u*_*D*_⟶*u*_0_ in *L*^2^(*ℝ*) and *u*_*D*_⇀*u*_0_ in *H*^*s*^(*ℝ*), we have *∂*_*x*_*u*_*D*_⇀*∂*_*x*_*u*_0_ in *H*^*s*−1^(*ℝ*) and *∂*_*x*_^2^*u*_*D*_⇀*∂*_*x*_^2^*u*_0_ in *H*^*s*−2^(*ℝ*) uniformly on [0, *T*_*s*_]. Observe that if *r* > 1/2, *f*_*n*_⇀*f* in *H*^*r*^(*ℝ*) and *g*_*n*_⇀*g* in *H*^*r*^(*ℝ*) then *f*_*n*_*g*_*n*_⇀*fg* in *H*^*r*^(*ℝ*). After, we have

(52) $$\begin{array}{c} u_{D}\left(u_{D}-\alpha \right)\left(u_{D}-1\right)⇀u_{0}\left(u_{0}-\alpha \right)\left(u_{0}-1\right)\text{ in }H^{s}\left(\mathbb{R}\right),\\ u_{D}^{n}\partial_{x}\left(u_{D}\right)⇀u_{0}^{n}\partial_{x}\left(u_{0}\right)\text{ in }H^{s-1}\left(\mathbb{R}\right), \end{array}$$

uniformly on [0, *T*_*s*_]. Thereby, taking the limit as *D*⟶0+ in (51), we obtain as follows:

(53) $$\begin{array}{c} {\left(u_{0}\left(t\right)\left|\varphi \right|\right)}_{s-2}={\left(\psi \left|\varphi \right|\right)}_{s-2}+\int_{0}^{t}{\left(-u_{0}\left(u_{0}-\alpha \right)\left(u_{0}-1\right)-\epsilon u_{0}^{n}\partial_{x}u_{0}\left|\varphi \right|\right)}_{s-2}d\tau . \end{array}$$

**Corollary 1 .**

Let *u*_0_ be as in the preceding theorem, then *u*_0_ ∈ *AC*([0, *T*_*s*_]; *H*^*s*−2^(*ℝ*)).

**Proof**

Since *t* ∈ [0, *T*_*s*_(*ψ*)] ↦ *u*(*u* − *α*)(*u* − 1)+*ϵu*^*n*^*u*_*x*_ is weakly continuous in *H*^*s*−2^(*ℝ*) and the Sobolev space is separable, then applying the Bochner–Pettis theorem, it is a strongly measurable function in *H*^*s*−2^(*ℝ*). Therefore,

(54) $$\begin{array}{c} \int_{0}^{t}\left(u\left(u-\alpha \right)\left(u-1\right)+\epsilon u^{n}u_{x}\right)d\tau , \end{array}$$

exists as a Bochner integral. So, from (53) we conclude that

(55) $$\begin{array}{c} u_{0}\left(t\right)=\psi +\int_{0}^{t}\left(u\left(u-\alpha \right)\left(u-1\right)+\epsilon u^{n}u_{x}\right)d\tau , \end{array}$$

and therefore, *u*_0_ ∈ *AC*([0, *T*_*s*_]; *H*^*s*−2^(*ℝ*)).

**Theorem 4 .**

Let *s* > 3/2 and *T* > 0 be fixed, *ψ*_*j*_ ∈ *H*^*s*^(*ℝ*), *j*=1,2, and *v*_*j*_ ∈ *C*([0, *T*]; *L*^2^(*ℝ*))∩*C*_*w*_([0, *T*]; *H*^*s*^(*ℝ*))∩*AC*([0, *T*]; *H*^*s*−2^(*ℝ*)) two weak sense solutions to (1) with *D*=0 such that *v*_*j*_(0)=*ψ*_*j*_, *j*=1,2. Then,

(56) $$\begin{array}{c} {\left\|v_{1}\left(t\right)-v_{2}\left(t\right)\right\|}_{0}\leq {\left\|\psi_{1}-\psi_{2}\right\|}_{0}e^{t\left(L_{0}\left(R,R\right)+\alpha \right)}, \end{array}$$

where *L*_0_ is as in the Proposition 7 and

(57) $$\begin{array}{c} R=\max\left\{\underset{t\in \left[0,T\right]}{\sup}{\left\|v_{1}\left(t\right)\right\|}_{s},\underset{t\in \left[0,T\right]}{\sup}{\left\|v_{2}\left(t\right)\right\|}_{s}\right\}. \end{array}$$

**Proof**

Let *w*(*t*)=*v*_1_(*t*) − *v*_2_(*t*). Since *s* > 3/2, we have *s* − 2 > −1/2 > 1 − *s* and 1 − *s* > −*s*, and also *H*^*s*−2^(*ℝ*)⟶*H*^−*s*^(*ℝ*). Using the fact that *w*(*t*) is real valued we have

(58) $$\begin{array}{c} \frac{{\left(w\left(t+h\right)\left|w\left(t+h\right)\right|\right)}_{0}-{\left(w\left(t\right)\left|w\left(t\right)\right|\right)}_{0}}{h}\\ ={\left(\frac{w\left(t+h\right)-w\left(t\right)}{h}\left|w\left(t+h\right)\right|\right)}_{0}+{\left(\frac{w\left(t+h\right)-w\left(t\right)}{h}\left|w\left(t\right)\right|\right)}_{0}\\ ={\langle \frac{w\left(t+h\right)-w\left(t\right)}{h}\left|w\left(t+h\right)\right|\rangle }_{s}+{\langle \frac{w\left(t+h\right)-w\left(t\right)}{h}\left|w\left(t\right)\right|\rangle }_{s}, \end{array}$$

where *t* ∈ [0, *T*] is fixed, *h* is such that *t*+*h* ∈ [0, *T*], and 〈·|·|〉_*s*_ is the *H*^*s*^ duality bracket. As *t* ∈ [0, *T*] ↦ *w*(*t*) ∈ *H*^*s*^(*ℝ*) is bounded and

(59) $$\begin{array}{c} \partial_{t}w\left(t\right)=\lim_{h⟶0}\frac{w\left(t+h\right)-w\left(t\right)}{h}=-\left(v_{1}\left(t\right)\left(v_{1}\left(t\right)-\alpha \right)\left(v_{1}\left(t\right)-1\right)-v_{2}\left(t\right)\left(v_{2}\left(t\right)-\alpha \right)\left(v_{2}\left(t\right)-1\right)\right)\\ -\epsilon \left(v_{1}{\left(t\right)}^{n}\partial_{x}v_{1}\left(t\right)-v_{2}{\left(t\right)}^{n}\partial_{x}v_{2}\left(t\right)\right), \end{array}$$

exists in the norm of *H*^*s*−2^(*ℝ*)⟶*H*^−*s*^(*ℝ*), from (58) and (59) we have as follows:

(60) $$\begin{array}{c} \partial_{t}{\left\|w\left(t\right)\right\|}_{0}^{2}\\ =-2{\langle v_{1}\left(t\right)\left(v_{1}\left(t\right)-\alpha \right)\left(v_{1}\left(t\right)-1\right)-v_{2}\left(t\right)\left(v_{2}\left(t\right)-\alpha \right)\left(v_{2}\left(t\right)-1\right)+\epsilon v_{1}{\left(t\right)}^{n}\partial_{x}v_{1}\left(t\right)-\epsilon v_{2}{\left(t\right)}^{n}\partial_{x}v_{2}\left(t\right)\left|w\left(t\right)\right|\rangle }_{s}\\ =-2\left(v_{1}\left(t\right)\left(v_{1}\left(t\right)-\alpha \right)\left(v_{1}\left(t\right)-1\right)-v_{2}\left(t\right)\left(v_{2}\left(t\right)-\alpha \right)\left(v_{2}\left(t\right)-1\right)+\epsilon v_{1}{\left(t\right)}^{n}\partial_{x}v_{1}\left(t\right){\left.-\epsilon v_{2}{\left(t\right)}^{n}\partial_{x}v_{2}\left(t\right)\left|w\left(t\right)\right|\right)}_{0}\right)\\ =-2{\left(v_{1}{\left(t\right)}^{3}-\left(\alpha +1\right)v_{1}{\left(t\right)}^{2}-\epsilon v_{1}{\left(t\right)}^{n}\partial_{x}v_{1}\left(t\right)-\left(v_{2}{\left(t\right)}^{3}-\left(\alpha +1\right)v_{2}{\left(t\right)}^{2}-\epsilon v_{2}{\left(t\right)}^{n}\partial_{x}v_{2}\left(t\right)\right)+\alpha \left(v_{1}\left(t\right)-v_{2}\left(t\right)\right)\left|w\left(t\right)\right|\right)}_{0}. \end{array}$$

From Proposition 7 and (60) we have as follows:

(61) $$\begin{array}{c} \partial_{t}{\left\|w\left(t\right)\right\|}_{0}^{2}\leq \left(L_{0}\left(R,R\right)+\alpha \right){\left\|v_{1}\left(t\right)-v_{2}\left(t\right)\right\|}_{0}^{2}, \end{array}$$

where *R* is given by (57). Applying Gronwall's inequality to (61), and we have proved the theorem.

**Theorem 5 .**

Let *ψ* ∈ *H*^*s*^(*ℝ*) with *s* > 3/2. Then, there exists a *T*_*s*_=*T*_*s*_(*ψ*) > 0 and a unique *u*_0_ ∈ *C*([0, *T*_*s*_]; *H*^*s*^(*ℝ*)) such that

(62) $$\begin{array}{c} \left\{\begin{array}{c} \partial_{t}u_{0}\left(t\right)+u_{0}\left(t\right)\left(u_{0}\left(t\right)-\alpha \right)\left(u_{0}\left(t\right)-1\right)+u_{0}{\left(t\right)}^{n}\partial_{x}u_{0}=0,\\ u_{0}\left(0\right)=\psi . \end{array}\right. \end{array}$$

**Proof**

From the previous results, there exists a unique solution of (62) in the class described in Theorem 4. Now, we will show that *u*_0_ ∈ *C*([0, *T*_*s*_]; *H*^*s*^(*ℝ*)).

Let *φ* ∈ *H*^*s*^(*ℝ*) be such that ‖*φ*‖_*s*_=1. Then, we have |(*u*_0_|*φ*|)_*s*_| ≤ ‖*u*_0_(*t*)‖_*s*_ ≤ *ρ*(*t*)^1/2^, for all *φ* ∈ *H*^*s*^(*ℝ*) and for all *t* ∈ [0, *T*_*s*_]. Additionally,

(63) $$\begin{array}{c} \left|{\left(\psi \left|\varphi \right|\right)}_{s}\right|=\lim_{t⟶0}+\left|{\left(u_{0}\left|\varphi \right|\right)}_{s}\right|=\underset{t⟶0}{\mathrm{liminf}}+\left|{\left(u_{0}\left|\varphi \right|\right)}_{s}\right|\leq \underset{t⟶0}{\mathrm{liminf}}+{\left\|u_{0}\left(t\right)\right\|}_{s}\\ \leq \underset{t⟶0}{\mathrm{limsup}}+{\left\|u_{0}\left(t\right)\right\|}_{s}\leq \underset{t⟶0}{\mathrm{limsup}}+\rho {\left(t\right)}^{1/2}={\left\|\psi \right\|}_{s}, \end{array}$$

for all *φ* ∈ *H*^*s*^(*ℝ*). As we have ‖*ψ*‖_*s*_=sup_‖*φ*‖_*s*__=1|(*ψ*|*φ*|)_*s*_|, then taking supremum over ‖*φ*‖_*s*_=1 in (63) we have liminf_*t*⟶0_+‖*u*_0_(*t*)‖_*s*_=limsup_*t*⟶0_+‖*u*_0_(*t*)‖_*s*_=‖*ψ*‖_*s*_, i.e., the limit of ‖*u*_0_(*t*)‖_*s*_ exists as *t*⟶0+ and lim_*t*⟶0_+‖*u*_0_(*t*)‖_*s*_=‖*ψ*‖_*s*_. Since *u*(*t*)⟶*ψ* weakly in *H*^*s*^(*ℝ*) as *t*⟶0+, it follows that lim_*t*⟶0_+*u*_0_(*t*)=*ψ* in the norm of *H*^*s*^(*ℝ*). Let *t*′ ∈ [0, *T*_*s*_) be fixed. Then, there exists $\tilde{T}>0$, with $T_{s}-t^{\prime }>\tilde{T}$, and a unique $v\in C_{w}\left(\left[0,\tilde{T}\right];H^{s}\left(\mathbb{R}\right)\right)\cap C_{w}^{1}\left(\left[0,\tilde{T}\right];H^{s-2}\left(\mathbb{R}\right)\right)$ satisfying *∂*_*t*_*v*(*t*)+*v*(*t*)(*v*(*t*) − *α*)(*v*(*t*) − 1)+*v*(*t*)^*n*^*∂*_*x*_*v*=0, with *v*(0)=*u*(*t*′). We have noticed that the uniqueness of solutions implies that *v*(*t*)=*u*_0_(*t*+*t*′), for $t\in \left[0,\tilde{T}\right]$. Since *v* is continuous from the right at *t*=0, then *u*_0_ is continuous from the right at *t*=*t*′. Now, let *t*′ ∈ (0, *T*] be fixed. Observe that the following problem

(64) $$\begin{array}{c} \left\{\begin{array}{c} -\partial_{t}w\left(t\right)+w\left(t\right)\left(w\left(t\right)-\alpha \right)\left(w\left(t\right)-1\right)-w{\left(t\right)}^{n}\partial_{x}w=0,\\ w\left(0\right)=\tilde{u}\left(t^{\prime }\right), \end{array}\right. \end{array}$$

has a unique solution *w*(*t*, *x*)=*u*_0_(*t*′ − *t*, −*x*) with $\tilde{u}\left(t^{\prime },x\right)=u_{0}\left(t^{\prime },-x\right)$, because the equation in problem (64) is similar to the equation in problem (1) with *D*=0 and it is easy to show similar results to those obtained for problem (1) with *D*=0, specially the uniqueness results.

In particular, for the problem (64) there are results analogous to Theorems 3 and 4. Therefore, since *w* is continuous from the right at *t*=0, then *u*_0_ is continuous from the left at *t*′. So, *u*_0_ ∈ *C*([0, *T*_*s*_]; *H*^*s*^(*ℝ*)). Moreover, we have *u*_0_(*u*_0_ − *α*)(*u*_0_ − 1)+*ϵu*_0_^*n*^*∂*_*x*_*u*_0_ ∈ *C*([0, *T*]; *H*^*s*−2^(*ℝ*)). From (55) we also conclude that *u*_0_ ∈ *C*^1^([0, *T*]; *H*^*s*−2^(*ℝ*)) and that it is the unique strong solution of (1) with *D*=0.

**Theorem 6 .**

Let *s* > 3/2, *ψ* ∈ *H*^*s*+1^(*ℝ*) and *u*_*D*_ ∈ *C*([0, *T*_*s*+1_(*ψ*)]; *H*^*s*+1^(*ℝ*))∩*C*^1^([0, *T*_*s*+1_(*ψ*)]; *H*^*s*−1^(*ℝ*)) be the corresponding solution of the problem (1) for *D* ≥ 0, defined in the interval [0, *T*_*s*+1_(*ψ*)] which is independent of *D*. Then, *u*_*D*_ can be extended, if necessary, to the interval [0, *T*_*s*_], with *ψ* viewed as an element of *H*^*s*^(*ℝ*).

**Proof**

Applying (30) with *r*=*s*+1 to obtain

(65) $$\begin{array}{c} \frac{d}{dt}{\left\|u_{D}\right\|}_{s+1}^{2}=2{\left(u_{D}\left|\partial u_{D}\left(t\right)\right|\right)}_{s+1}\\ =2\left[-D{\left\|\partial_{x}u_{D}\right\|}_{s+1}^{2}-{\left(u_{D}\left|u_{D}\left(u_{D}-\alpha \right)\left(u_{D}-1\right)\right|\right)}_{s+1}-\epsilon {\left(u_{D}\left|u_{D}^{n}\partial_{x}u_{D}\right|\right)}_{s+1}\right]\\ \leq 2C_{s}\left({\left\|u_{D}^{3}\right\|}_{s+1}{\left\|u_{D}\right\|}_{s+1}+\left(\alpha +1\right){\left\|u_{D}^{2}\right\|}_{s+1}{\left\|u_{D}\right\|}_{s+1}+{\left\|u_{D}\right\|}_{s}^{n}{\left\|u_{D}\right\|}_{s+1}^{2}\right). \end{array}$$

Now, from inequality (3.12) and Theorem 3.2 in [14], we obtain as follows:

(66) $$\begin{array}{c} {\left\|u_{D}^{2}\right\|}_{s+1}\leq 2{\left\|u_{D}\right\|}_{L^{\infty }}{\left\|u_{D}\right\|}_{s+1}\leq {\left\|u_{D}\right\|}_{s}{\left\|u_{D}\right\|}_{s+1}, \end{array}$$

(67) $$\begin{array}{c} {\left\|u_{D}^{3}\right\|}_{s+1}\leq \left({\left\|u_{D}\right\|}_{L^{\infty }}{\left\|u_{D}^{2}\right\|}_{s+1}+{\left\|u_{D}^{2}\right\|}_{L^{\infty }}{\left\|u_{D}\right\|}_{s+1}\right)\\ \leq C{\left\|u_{D}\right\|}_{s}^{2}{\left\|u_{D}\right\|}_{s+1}. \end{array}$$

Therefore, using (66), (67) in (65), we have as follows:

(68) $$\begin{array}{c} \frac{d}{dt}{\left\|u_{D}\left(t\right)\right\|}_{s+1}^{2}\leq 2C^{\prime }\left({\left\|u_{D}\left(t\right)\right\|}_{s}^{2}+\left(\alpha +1\right){\left\|u_{D}\left(t\right)\right\|}_{s}+{\left\|u_{D}\left(t\right)\right\|}_{s}^{n}\right){\left\|u_{D}\left(t\right)\right\|}_{s+1}^{2}, \end{array}$$

and integrating from 0 to *t*, we have as follows:

(69) $$\begin{array}{c} {\left\|u_{D}\left(t\right)\right\|}_{s+1}^{2}\leq {\left\|\psi \right\|}_{s+1}^{2}+2C^{\prime }\int_{0}^{t}\left({\left\|u_{D}\left(\tau \right)\right\|}_{s}^{2}+\left(\alpha +1\right){\left\|u_{D}\left(\tau \right)\right\|}_{s}+{\left\|u_{D}\left(\tau \right)\right\|}_{s}^{n}\right){\left\|u_{D}\left(\tau \right)\right\|}_{s+1}^{2}d\tau , \end{array}$$

and applying the Gronwall's inequality to obtain

(70) $$\begin{array}{c} {\left\|u_{D}\left(t\right)\right\|}_{s+1}^{2}\leq {\left\|\psi \right\|}_{s+1}^{2}\exp\left(2C^{\prime }\int_{0}^{t}\left({\left\|u_{D}\left(\tau \right)\right\|}_{s}^{2}+\left(\alpha +1\right){\left\|u_{D}\left(\tau \right)\right\|}_{s}+{\left\|u_{D}\left(\tau \right)\right\|}_{s}^{n}\right)d\tau \right). \end{array}$$

Observe that on the right-hand side of (70) is well-defined for *t* ∈ [0, *T*_*s*_(*ψ*)] and therefore we can extend (if necessary) *u*=*u*(*t*) to [0, *T*_*s*_(*ψ*)] as a solution in *H*^*s*+1^(*ℝ*). Thus, we conclude that *T*_*s*_(*ψ*) ≤ *T*_*s*+1_(*ψ*). So, *u*_*D*_ ∈ *C*([0, *T*_*s*_(*ψ*)]; *H*^*s*+1^(*ℝ*)) for *D* > 0. From (70) also we have that

(71) $$\begin{array}{c} {\left\|u_{D}\left(t\right)\right\|}_{s+1}^{2}\leq {\left\|\psi \right\|}_{s+1}^{2}\exp\left(2C^{\prime }\left(M^{2}+\left(\alpha +1\right)M+M^{n}\right)T_{s}\left(\psi \right)\right). \end{array}$$

Observe that the last inequality is independent of *D* > 0 and since *u*_*D*_ weakly converges and uniformly to *u*_0_ in *H*^*s*+1^(*ℝ*), then we have *u*_0_ ∈ *C*([0, *T*_*s*_(*ψ*)]; *H*^*s*+1^(*ℝ*)).

Following Lemma 5 in [15] we have the next lemma.

**Lemma 4 .**

Let *s* > 3/2. For *ψ* ∈ *H*^*s*^(*ℝ*) and *τ* > 0, we define

(72) $$\begin{array}{c} \psi^{\tau }=\exp\left(-\tau {\left(1-\partial_{x}^{2}\right)}^{s/2}\right)\psi ={\left(\hat{\psi }\left(\cdot \right)\exp{\left(-\tau \left(1+{\left|\cdot \right|}^{2}\right)\right)}^{s/2}\right)}^{\vee }. \end{array}$$

Then, lim_*τ*⟶0_+‖*ψ*^*τ*^ − *ψ*‖_*s*_=0, and there exists a constant *C*=*C*(*s*) such that

(73) $$\begin{array}{c} {\left\|\psi^{\tau }\right\|}_{s+1}\leq C{\left(1+{\left(\frac{1}{\tau s}\right)}^{2/s}\right)}^{1/2}{\left\|\psi \right\|}_{s},\\ {\left\|\psi^{\tau }-\psi^{\theta }\right\|}_{0}\leq \left|\tau -\theta \right|{\left\|\psi \right\|}_{s}. \end{array}$$

Moreover, lim_*τ*⟶0_+‖*ψ*^*τ*^ − *ψ*‖_*s*_=0 uniformly on compact subsets of *H*^*s*^(*ℝ*).

**Proof**

Notice that ${\left\|\psi^{\tau }-\psi \right\|}_{s}^{2}=\int_{\mathbb{R}}{\left(1+\xi^{2}\right)}^{s}{\left|e^{-\tau {\left(1+\xi^{2}\right)}^{s/2}}-1\right|}^{2}{\left|\hat{\psi }\left(\xi \right)\right|}^{2}d\xi .$ Then, using Lebesque's dominated convergence theorem, we obtain lim_*τ*⟶0_+‖*ψ*^*τ*^ − *ψ*‖_*s*_=0. Now, to prove the uniformity on compact subsets, it is enough to show that *ψ*_*n*_⟶*ψ* in *H*^*s*^(*ℝ*) implies lim_*τ*⟶0_+‖*ψ*_*n*_^*τ*^ − *ψ*_*n*_‖_*s*_=0 uniformly for *n*=1,2,…, since sequential compactness is equivalent to compactness in metric spaces. Thus, observe that

(74) $$\begin{array}{c} {\left\|\psi_{n}^{\tau }-\psi^{\tau }\right\|}_{s}^{2}=\int_{\mathbb{R}}{\left(1+\xi^{2}\right)}^{s}e^{-2\tau {\left(1+\xi^{2}\right)}^{s/2}}{\left|{\hat{\psi }}_{n}\left(\xi \right)-\hat{\psi }\left(\xi \right)\right|}^{2}d\xi \\ \leq \int_{\mathbb{R}}{\left(1+\xi^{2}\right)}^{s}{\left|{\hat{\psi }}_{n}\left(\xi \right)-\hat{\psi }\left(\xi \right)\right|}^{2}d\xi ={\left\|\psi_{n}-\psi \right\|}_{s}^{2}. \end{array}$$

Let *ϵ* > 0 be given and choose *N* such that if *n* ≥ *N*, then ‖*ψ*_*n*_ − *ψ*‖_*s*_ < (1/3)*ϵ*. Thus, for *τ*_0_ > 0 small enough that 0 < *τ* < *τ*_0_ we have

(75) $$\begin{array}{c} \left\|\psi_{n}^{\tau }-\psi_{n}\right\|<\epsilon , \end{array}$$

for 1 ≤ *n* ≤ *N*. Now, if *n* ≥ *N* then we have

(76) $$\begin{array}{c} {\left\|\psi_{n}^{\tau }-\psi_{n}\right\|}_{s}\leq {\left\|\psi_{n}^{\tau }-\psi^{\tau }\right\|}_{s}+{\left\|\psi^{\tau }-\psi \right\|}_{s}+{\left\|\psi -\psi_{n}\right\|}_{s}\\ \leq {\left\|\psi_{n}-\psi \right\|}_{s}+{\left\|\psi^{\tau }-\psi \right\|}_{s}+{\left\|\psi -\psi_{n}\right\|}_{s}<\epsilon . \end{array}$$

Hence (75) holds for all *n*.

On the other hand, we have

(77) $$\begin{array}{c} {\left\|\psi^{\tau }\right\|}_{s+1}^{2}=\int_{\mathbb{R}}{\left(1+\xi^{2}\right)}^{s+1}e^{-2\tau {\left(1+\xi^{2}\right)}^{s/2}}{\left|\hat{\psi }\left(\xi \right)\right|}^{2}d\xi \leq \left(1+\max_{\xi \in \mathbb{R}}\left(\xi^{2}e^{-2\tau {\left(1+\xi^{2}\right)}^{s/2}}\right)\right){\left\|\psi \right\|}_{s}^{2}\\ \leq C\left(1+{\left(\frac{1}{\tau s}\right)}^{2/s}\right). \end{array}$$

Finally, using the mean value theorem, |*e*^−*τ*(1+*ξ*^2^)^*s*/2^^ − *e*^−*τ*(1+*ξ*^2^)^*s*/2^^| ≤ |*τ* − *θ*|(1+*ξ*^2^)^*s*/2^, and then we have

(78) $$\begin{array}{c} {\left\|\psi^{\tau }-\psi^{\theta }\right\|}_{0}^{2}=\int_{\mathbb{R}}{\left|\psi^{\tau }\left(x\right)-\psi^{\theta }\left(x\right)\right|}^{2}dx\\ =\int_{\mathbb{R}}{\left|e^{-\tau {\left(1+\xi^{2}\right)}^{s/2}}-e^{-\tau {\left(1+\xi^{2}\right)}^{s/2}}\right|}^{2}{\left|\hat{\psi }\left(\xi \right)\right|}^{2}d\xi \leq {\left|\tau -\theta \right|}^{2}{\left\|\psi \right\|}_{s}^{2}. \end{array}$$

The proof is complete.

**Proposition 8 .**

Let *s* > 3/2, *ψ* ∈ *H*^*s*^(*ℝ*), *ψ*^*τ*^ (for *τ* > 0) be as in the preceding lemma. If *u*_0_^*τ*^ is solution of the problem (62) with *u*_0_^*τ*^(0)=*ψ*^*τ*^, for all *τ* > 0, then there are constants *C*=*C*(*s*, ‖*ψ*‖_*s*_, *T*) > 0 and *η*=*η*(*s*) ∈ (0,1) such that

(79) $$\begin{array}{c} {\left\|u_{0}^{\tau }-u_{0}^{\theta }\right\|}_{s}^{2}\leq C\left[{\left\|\psi^{\tau }-\psi^{\theta }\right\|}_{s}^{2}+\tau^{1-\eta }\right], \end{array}$$

for *τ* sufficiently small and 0 ≤ *θ* ≤ 2*τ*.

**Proof**

Let *τ*_0_ > 0 be such that *u*_0_^*τ*^(*t*) is well-define in [0, *T*] for all 0 < *τ* ≤ *τ*_0_. Then,

(80a) $$\begin{array}{c} \frac{1}{2}\partial_{t}{\left\|u_{0}^{\tau }-u_{0}^{\theta }\right\|}_{s}^{2}={\left(u_{0}^{\tau }-u_{0}^{\theta }\left|u_{0}^{\tau }\left(u_{0}^{\tau }-\alpha \right)\left(u_{0}^{\tau }-1\right)-u_{0}^{\theta }\left(u_{0}^{\theta }-\alpha \right)\left(u_{0}^{\theta }-1\right)\right|\right)}_{s}\\ \leq \left|{\left(u_{0}^{\tau }-u_{0}^{\theta }\left|u_{0}^{\tau }\left(u_{0}^{\tau }-\alpha \right)\left(u_{0}^{\tau }-1\right)-u_{0}^{\theta }\left(u_{0}^{\theta }-\alpha \right)\left(u_{0}^{\theta }-1\right)\right|\right)}_{s}\right|, \end{array}$$

(80b) $$\begin{array}{c} +\epsilon \left|{\left(u_{0}^{\tau }-u_{0}^{\theta }\left|{\left(u_{0}^{\theta }\right)}^{n}\partial_{x}\left(u_{0}^{\tau }-u_{0}^{\theta }\right)\right|\right)}_{s}\right|, \end{array}$$

(80c) $$\begin{array}{c} +\epsilon \left|{\left(u_{0}^{\tau }-u_{0}^{\theta }\left|\left({\left(u_{0}^{\tau }\right)}^{n}-{\left(u_{0}^{\theta }\right)}^{n}\right)\partial_{x}u_{0}^{\tau }\right|\right)}_{s}\right|. \end{array}$$

Now, the right-hand side of the inequality (3) will be estimated.

First, we will estimate (80a). Applying the Cauchy–Schwartz inequality to (80a) we have

(81) $$\begin{array}{c} \left|{\left(u_{0}^{\tau }-u_{0}^{\theta }\left|u_{0}^{\tau }\left(u_{0}^{\tau }-\alpha \right)\left(u_{0}^{\tau }-1\right)-u_{0}^{\theta }\left(u_{0}^{\theta }-\alpha \right)\left(u_{0}^{\theta }-1\right)\right|\right)}_{s}\right|\leq \tilde{L}\left(\left\|u_{0}^{\tau }\right\|,\left\|u_{0}^{\theta }\right\|\right){\left\|u_{0}^{\tau }-u_{0}^{\theta }\right\|}_{s}^{2}\\ \leq \tilde{C}{\left\|u_{0}^{\tau }-u_{0}^{\theta }\right\|}_{s}^{2}, \end{array}$$

where $\tilde{L}\left(x,y\right)=x^{2}+xy+y^{2}+\left(\alpha +1\right)\left(x+y\right)+\alpha$ and $\tilde{C}=\tilde{L}\left(M,M\right)$.

Now, we will estimate (80b). Observe that

(82) $$\begin{array}{c} \left|{\left(u_{0}^{\tau }-u_{0}^{\theta }\left|{\left(u_{0}^{\theta }\right)}^{n}\partial_{x}\left(u_{0}^{\tau }-u_{0}^{\theta }\right)\right|\right)}_{s}\right|\leq C{\left\|\partial_{x}{\left(u_{0}^{\theta }\right)}^{n}\right\|}_{s-1}{\left\|u_{0}^{\tau }-u_{0}^{\theta }\right\|}_{s}^{2}\\ \leq C{\left\|u_{0}^{\theta }\right\|}_{s}^{n}{\left\|u_{0}^{\tau }-u_{0}^{\theta }\right\|}_{s}^{2}\leq CM^{n}{\left\|u_{0}^{\tau }-u_{0}^{\theta }\right\|}_{s}^{2}. \end{array}$$

Finally, we estimate (80c). As *s* > 3/2, there is *s*_0_ such that 3/2 < *s*_0_+1 < *s*. From the Cauchy–Schwartz inequality, we obtain

(83) $$\begin{array}{c} \left|{\left(u_{0}^{\tau }-u_{0}^{\theta }\left|\left({\left(u_{0}^{\tau }\right)}^{n}-{\left(u_{0}^{\theta }\right)}^{n}\right)\partial_{x}u_{0}^{\tau }\right|\right)}_{s}\right|\leq {\left\|u_{0}^{\tau }-u_{0}^{\theta }\right\|}_{s}{\left\|\left({\left(u_{0}^{\tau }\right)}^{n}-{\left(u_{0}^{\theta }\right)}^{n}\right)\partial_{x}u_{0}^{\tau }\right\|}_{s}\\ \leq {\left\|u_{0}^{\tau }-u_{0}^{\theta }\right\|}_{s}C\left({\left\|{\left(u_{0}^{\tau }\right)}^{n}-{\left(u_{0}^{\theta }\right)}^{n}\right\|}_{s}{\left\|u_{0}^{\tau }\right\|}_{s}+{\left\|{\left(u_{0}^{\tau }\right)}^{n}-{\left(u_{0}^{\theta }\right)}^{n}\right\|}_{s_{0}}{\left\|u_{0}^{\tau }\right\|}_{s+1}\right). \end{array}$$

Now, we will estimate each term on the right-hand side of the last inequality. First, observe that

(84) $$\begin{array}{c} {\left\|{\left(u_{0}^{\tau }\right)}^{n}-{\left(u_{0}^{\theta }\right)}^{n}\right\|}_{s}{\left\|u_{0}^{\tau }\right\|}_{s}\leq {\left\|u_{0}^{\tau }-u_{0}^{\theta }\right\|}_{s}{\left\|q\left(u_{0}^{\tau },u_{0}^{\theta }\right)\right\|}_{s}{\left\|u_{0}^{\tau }\right\|}_{s}\leq CM{\left\|u_{0}^{\tau }-u_{0}^{\theta }\right\|}_{s}, \end{array}$$

where *q* is defined in the proof of Proposition 7.

We also estimate ‖(*u*_0_^*τ*^)^*n*^ − (*u*_0_^*θ*^)^*n*^‖_*s*_0__‖*u*_0_^*τ*^‖_*s*+1_. From Lemma 4 and the inequality (71), we have

(85) $$\begin{array}{c} {\left\|u_{0}^{\tau }\right\|}_{s+1}\leq {\left\|\psi^{\tau }\right\|}_{s+1}e^{CT_{s}\left(M^{2}+\left(\alpha +1\right)M+M^{n}\right)}\leq c{\left\|\psi \right\|}_{s}\tau^{-\left(1/s\right)}, \end{array}$$

for all *τ* ≤ *τ*_0_.

From Lemma 4, we have

(86) $$\begin{array}{c} {\left\|{\left(u_{0}^{\tau }\right)}^{n}-{\left(u_{0}^{\theta }\right)}^{n}\right\|}_{s_{0}}\leq {\left\|q\left(u_{0}^{\tau },u_{0}^{\theta }\right)\right\|}_{s_{0}}{\left\|u_{0}^{\tau }-u_{0}^{\theta }\right\|}_{s_{0}}\leq C{\left\|u_{0}^{\tau }-u_{0}^{\theta }\right\|}_{s}^{\varrho }{\left\|u_{0}^{\tau }-u_{0}^{\theta }\right\|}_{0}^{1-\varrho }\\ \leq 2{\left(CM\right)}^{\varrho }{\left\|u_{0}^{\tau }-u_{0}^{\theta }\right\|}_{0}^{1-\varrho }, \end{array}$$

where ϱ=(*s*_0_/*s*). To estimate the term ‖*u*_0_^*τ*^ − *u*_0_^*θ*^‖_0_, observe that

(87) $$\begin{array}{c} \partial_{t}{\left\|u_{0}^{\tau }-u_{0}^{\theta }\right\|}_{0}^{2}=2{\left(u_{0}^{\tau }-u_{0}^{\theta }\left|u_{0}^{\tau }\left(u_{0}^{\tau }-\alpha \right)\left(u_{0}^{\tau }-1\right)-u_{0}^{\theta }\left(u_{0}^{\theta }-\alpha \right)\left(u_{0}^{\theta }-1\right)\right|\right)}_{0}\\ +2\epsilon {\left(u_{0}^{\tau }-u_{0}^{\theta }\left|{\left(u_{0}^{\tau }\right)}^{n}\partial_{x}u_{0}^{\tau }-{\left(u_{0}^{\theta }\right)}^{n}\partial_{x}u_{0}^{\theta }\right|\right)}_{0}\leq C_{1}{\left\|u_{0}^{\tau }-u_{0}^{\theta }\right\|}_{0}^{2}+\frac{1}{n+1}{\left\|\tilde{q}\left(u_{0}^{\tau },u_{0}^{\theta }\right)\right\|}_{L^{\infty }}{\left\|u_{0}^{\tau }-u_{0}^{\theta }\right\|}_{0}^{2}\leq C{\left\|u_{0}^{\tau }-u_{0}^{\theta }\right\|}_{0}^{2}, \end{array}$$

where $\tilde{q}\left(u,v\right)=\sum_{j=0}^{n}u^{j}v^{n-j}$, and from Gronwall inequality we have as follows:

(88) $$\begin{array}{c} {\left\|u_{0}^{\tau }-u_{0}^{\theta }\right\|}_{0}^{2}\leq C{\left\|\psi^{\tau }-\psi^{\theta }\right\|}_{0}^{2}. \end{array}$$

From Lemma 4,

(89) $$\begin{array}{c} {\left\|{\left(u_{0}^{\tau }\right)}^{n}-{\left(u_{0}^{\theta }\right)}^{n}\right\|}_{s_{0}}{\left\|u_{0}^{\tau }\right\|}_{s+1}\leq C{\left\|\psi^{\tau }-\psi^{\theta }\right\|}_{0}^{1-\left(s_{0}/s\right)}{\left\|\psi \right\|}_{s}\tau^{-\left(1/s\right)}\\ \leq C{\left|\tau -\theta \right|}^{1-\left(s_{0}/s\right)}{\left\|\psi \right\|}_{s}^{1-\left(s_{0}/s\right)}{\left\|\psi \right\|}_{s}\tau^{-\left(1/s\right)}\\ \leq C{\left\|\psi \right\|}_{s}^{2-\left(s_{0}/s\right)}\tau^{1-\left(s_{0}+1/s\right)}, \end{array}$$

for 0 ≤ *θ* ≤ *τ*. Therefore, we have

(90) $$\begin{array}{c} {\left\|u_{0}^{\tau }-u_{0}^{\theta }\right\|}_{s}{\left\|{\left(u_{0}^{\tau }\right)}^{n}-{\left(u_{0}^{\theta }\right)}^{n}\right\|}_{s_{0}}{\left\|u_{0}^{\tau }\right\|}_{s+1}\leq 2MC{\left\|\psi \right\|}_{s}^{2-\left(s_{0}/s\right)}\tau^{1-\left(s_{0}+1/s\right)}, \end{array}$$

and the term (80c) is bounded for

(91) $$\begin{array}{c} \left|{\left(u_{0}^{\tau }-u_{0}^{\theta }\left|\left({\left(u_{0}^{\tau }\right)}^{n}-{\left(u_{0}^{\theta }\right)}^{n}\right)\partial_{x}u_{0}^{\tau }\right|\right)}_{s}\right|\leq C\left({\left\|u_{0}^{\tau }-u_{0}^{\theta }\right\|}_{s}^{2}+\tau^{1-\left(s_{0}+1/s\right)}\right). \end{array}$$

Of the bounds that were found for (80a), (80b), (80c) we conclude that

(92) $$\begin{array}{c} \partial_{t}{\left\|u_{0}^{\tau }-u_{0}^{\theta }\right\|}_{s}^{2}\leq \tilde{\tilde{C}}\left[{\left\|u_{0}^{\tau }-u_{0}^{\theta }\right\|}_{s}^{2}+\tau^{1-\left(s_{0}+1/s\right)}\right], \end{array}$$

and using Gronwall inequality, we obtain (79).

The following corollary follows immediately from Proposition 8 and Lemma 4.

**Corollary 2 .**

Let *F* be a compact subset in *H*^*s*^(*ℝ*). Suppose that *ψ* ∈ *F*, *ψ*^*τ*^ and *u*_0_^*τ*^ are defined as in the preceding result. Then, *u*_0_^*τ*^ converges uniformly to *u*_0_, for all *t* ∈ [0, *T*], as *τ*⟶0+.

**Theorem 7 .**

The map *ψ* ↦ *u*_0_ is continuous in the following sense: let *ψ*_*j*_ ∈ *H*^*s*^(*ℝ*), *j*=1,2,3,… such that *ψ*_*j*_⟶*ψ* in *H*^*s*^(*ℝ*) and *u*_0,*j*_ ∈ *C*((0, *T*_*s*,*j*_); *H*^*s*^(*ℝ*))∩*C*^1^((0, *T*_*s*,*j*_]; *H*^*s*−2^(*ℝ*)) are the corresponding solutions of the problem (62) with initial condition *u*_0,*j*_(0)=*ψ*_*j*_. Let *T* ∈ (0, *T*_*s*,*∞*_). Then, there exists a positive integer *N*_0_=*N*_0_(*s*, *ψ*) such that *T*_*s*,*j*_ ≥ *T* for all *j* ≥ *N*_0_ and

(93) $$\begin{array}{c} \lim_{j⟶\infty }\sup_{\left[0,T\right]}{\left\|u_{0,j}\left(t\right)-u_{0}\left(t\right)\right\|}_{s}=0. \end{array}$$

**Proof**

Consider *ψ* ∈ *H*^*s*^(*ℝ*) and let {*ψ*_*j*_}_*j*∈*ℕ*_ be a sequence in *H*^*s*^(*ℝ*) such that converges to *ψ*. Suppose that *u*_0_, *u*_0,*j*_, *u*_0_^*τ*^, *u*_0,*j*_^*τ*^ are the corresponding solutions of (62) with initial values *ψ*, *ψ*_*j*_, *ψ*^*τ*^, *ψ*_*j*_^*τ*^, respectively. As *ψ*_*j*_⟶*ψ*, from Corollary 2 we have that *u*_0,*j*_^*τ*^ converges uniformly to *u*_0,*j*_ and *u*_0_^*τ*^ converges uniformly to *u*_0_, as *τ*⟶0+. Thus, given *ϵ* > 0, for *τ* sufficiently small, we have

(94) $$\begin{array}{c} {\left\|u_{0,j}-u_{0}\right\|}_{s}={\left\|u_{0,j}-u_{0,j}^{\tau }+u_{0}^{\tau }-u_{0}+u_{0,j}^{\tau }-u_{0}^{\tau }\right\|}_{s}<2\epsilon +{\left\|u_{0,j}^{\tau }-u_{0}^{\tau }\right\|}_{s}, \end{array}$$

for all *t* ∈ [0, *T*]. Now, we will show that ‖*u*_0,*j*_^*τ*^ − *u*_0_^*τ*^‖_*s*_ converges uniformly to zero, as *j*⟶*∞*. In fact, from Lemma 3 and the Cauchy–Schwartz inequality, we have as follows:

(95) $$\begin{array}{c} \partial_{t}{\left\|u_{0,j}^{\tau }-u_{0}^{\tau }\right\|}_{s}^{2}=2{\left(u_{0,j}^{\tau }-u_{0}^{\tau }\left|u_{0,j}^{\tau }\left(u_{0,j}^{\tau }-\alpha \right)\left(u_{0,j}^{\tau }-1\right)-u_{0}^{\tau }\left(u_{0}^{\tau }-\alpha \right)\left(u_{0}^{\tau }-1\right)\right|\right)}_{s}\\ +2\epsilon {\left(u_{0,j}^{\tau }-u_{0}^{\tau }\left|{\left(u_{0,j}^{\tau }\right)}^{n}\partial_{x}u_{0,j}^{\tau }-{\left(u_{0}^{\tau }\right)}^{n}\partial_{x}u_{0}^{\tau }\right|\right)}_{s}\\ \leq C\left(\tilde{L}\left({\left\|u_{0,j}^{\tau }\right\|}_{s},{\left\|u_{0}^{\tau }\right\|}_{s}\right)+{\left\|u_{0}^{\tau }\right\|}_{s+1}^{n}+q\left({\left\|u_{0,j}^{\tau }\right\|}_{s},{\left\|u_{0}^{\tau }\right\|}_{s}\right)\right){\left\|u_{0,j}^{\tau }-u_{0}^{\tau }\right\|}_{s}^{2}. \end{array}$$

Applying Gronwall inequality to the last relation and the fact that ‖*ψ*_*j*_^*τ*^ − *ψ*^*τ*^‖_*s*_ ≤ ‖*ψ*_*j*_ − *ψ*‖_*s*_, we have

(96) $$\begin{array}{c} {\left\|u_{0,j}^{\tau }-u_{0}^{\tau }\right\|}_{s}^{2}\leq {\left\|\psi_{j}-\psi \right\|}_{s}^{2}\exp\left(c\int_{0}^{T}\left(\tilde{L}\left({\left\|u_{0,j}^{\tau }\right\|}_{s},{\left\|u_{0}^{\tau }\right\|}_{s}\right)+{\left\|u_{0}^{\tau }\right\|}_{s+1}^{n}+q\left({\left\|u_{0,j}^{\tau }\right\|}_{s},{\left\|u_{0}^{\tau }\right\|}_{s}\right)\right)d\tau \right). \end{array}$$

Therefore, for *j* sufficiently large, we have ‖*u*_0,*j*_ − *u*_0_‖_*s*_ ≤ *ϵ*, for all *t* ∈ [0, *T*].

Finally, the results obtained above can be summarized as follows:

**Theorem 8 .**

Let *s* > 3/2. For *D*=0, the problem (1) is locally well-posed in *H*^*s*^(*ℝ*).

## Data Availability

No data were used to support this study.
